# Dynamics of soliton propagation: bifurcation, chaos, and quantitative insights into the modified Camassa–Holm equation

**DOI:** 10.1038/s41598-026-37010-2

**Published:** 2026-02-06

**Authors:** Md. Nur Alam, Shams Forruque Ahmed, Hajar F. Ismael, Mitiku Daba Firdi, Irfan Anjum Badruddin, Syed Javed

**Affiliations:** 1 School of Mathematical Sciences, Sunway University, Bandar Sunway, 47500 Petaling Jaya, Selangor Darul Ehsan Malaysia; 2 Department of Mathematics, Pabna University of Science and Technology, Pabna, 6600 Bangladesh; 3 Department of Mathematics, Saveetha School of Engineering, Saveetha Institute of Medical and Technical Sciences, Chennai, 602105 Tamil Nadu India; 4 Miyan Research Institute, International University of Business Agriculture and Technology, Dhaka, 1230 Bangladesh; 5 Department of Mathematics, College of Science, University of Zakho, Zakho, 42002 Iraq; 6 Department of Applied Mathematics, Adama Science & Technology University, 1888 Adama, Ethiopia; 7 Mechanical Engineering Department, College of Engineering, King Khalid University, 61421 Abha, Saudi Arabia

**Keywords:** Camassa–Holm equation, Modified $(G{\prime}/G)$-expansion method, Soliton solutions, Quasi-periodic wave, Dynamical system, Lyapunov exponent, Bifurcation and phase portrait, Chaotic nature, Nonlinear differential equations, Mathematical physics, Engineering, Mathematics and computing, Physics

## Abstract

The modified Camassa–Holm (MCH) equation is a significant mathematical model for describing nonlinear wave phenomena, especially in shallow water dynamics and related physical systems. Although various analytical techniques have been applied to such nonlinear equations, many difficulties have arisen in producing a wide variety of exact and structurally rich solutions. This study addresses this gap by employing the modified (G′/G)-expansion (MG′/GE) method to construct an extensive range of exact traveling wave solutions for the MCH framework, such as trigonometric, hyperbolic, and rational solutions. Numerous waveforms, including single singular, double singular, multiple bright, multiple dark, multiple singular, and singular solitons, have been found to have solutions for the MCH framework. These waveforms have numerous applications in applied sciences and engineering. The structural properties and propagation dynamics of the resulting solutions are successfully depicted by graphics such as 3D, contour, density, 2D time-evolution, and 3D revolving plots. Compared to other existing approaches, such as the sine–cosine method and the tanh method, the MG’/GE approach is substantially more accurate and adaptable. The MG’/GE technique’s durability and computing efficiency allow it to generate precise findings straightforwardly. Its broad variety of applications in nonlinear system analysis is further highlighted by its expansion to fractional-order equations. In addition to laying the foundation for future research on traveling wave phenomena in many scientific domains, the current study presents an analytical scheme for both classical and fractional nonlinear evolution equations (NLEEs).

## Introduction

### Background of this research work

The study of soliton solutions in nonlinear evolution equations (NLEEs) is a rapidly growing research subject because it plays an indispensable role in the study of nonlinear dynamics in a wide variety of fields, including plasma physics, fluid mechanics, ocean engineering, optical fiber communication, and mechanical systems^1^. Ever since the original observation by Russell^2^ and the original work of Zabusky and Kruskal^3^, solitons have been known to retain shape and speed throughout long distances, even after interaction. It is this property that enables soliton theory to be used to reduce the destructive ocean waves and allow the transmission of data without distortion. It has been demonstrated in many studies^4–9^ that NLEEs are efficient to describe the nonlinear nature of natural phenomena and provide important information on the stability of a system, bifurcation, and wave propagation. Even though various methods of analysis have been developed, some of them do not universally apply to NLEEs. It requires the development of stronger and more universal tools to study soliton structures and nonlinear dynamical aspects in more complicated systems of the real world.

Analytical and integral-based approaches to building precise solutions to NLEEs have been created and widely used by researchers. A brief overview of the main techniques described in the literature, with a focus on their theoretical basis, implementation schemes, and their ability to obtain precise soliton and traveling wave solutions, is presented in this section. Among others, notable methods are the modified extended tanh expansion method^10^, the $\left( {G^{\prime } /G^{2} } \right)$-expansion function method^10^, the new Kudryashov method^11^, the Riccati equation method^11^, the KumarMalik method^11^, the modified auxiliary equation method^12^, the generalized projective Riccati equation method^12^, the new sub-equation method^13^, and the modified Khater method^13^, and the ${\Phi }^{6}$-model expansion method^14^.

### Literature review and formulation of the governing model

Perhaps the most important NLEE in the analysis of the dynamics of shallow water waves is the so-called Camassa-Holm (CH) equation, introduced by Camassa and Holm^15^. The CH equation is a unidirectional propagation model of waves derived as a bi-Hamiltonian approximation of nonlinear and dispersive wave propagation and is more accurate than classical models, e.g., the Kortewegde Vries (KdV) equation:

1 $${P}_{t}+2K{P}_{x}-{P}_{xxt}+3P{P}_{x}-2{P}_{x}{P}_{xx}-P{P}_{xx}=0.$$

When its dispersion parameter $K$ is positive, the CH equation has smooth solitary-wave solutions that are in close correspondence with real world profiles of shallow water waves. An especially striking aspect of the CH equation is that a peakon solution occurs when the parameter $K$ of the equation has a value of zero, that is, the solitary wave of the equation has a sharp crest and a slope discontinuity. These peaked solitons, which are not present in the KdV regime, illustrate the capability of the CH equation to model wave breaking and sharp fronted waves.

Due to its rich mathematical form, integrability, and physical applicability, the CH equation has been comprehensively modified and generalized by many researchers and physicists to fit a wide range of nonlinear wave behavioral phenomena. These extensions are still used today to provide a profound understanding of fluid dynamics, nonlinear wave theory, and the larger family of shallow water models. An inverse scattering method was used by Lundmark and Szmigielski^16^ to obtain multi-peakon solutions of the Degasperis-Procesi (DP) equation, extending the results of CH peakons and providing important information about the dynamics of peakons and their integrability. It was demonstrated that the DP equation is strongly dispersive in the sense that smooth solutions propagate at an infinite speed, immediately losing compact support (as pointed out by Mustafa^17^). Wazwaz^18^ came up with solitary wave solutions of modified DP and CH equations and demonstrated that the modifications convert the multi-peakon solutions to bell-shaped solitons. The study used soliton solutions using only tanh and sine–cosine approaches. Wazwaz^18^ offers the equations of the family in the following form:

2 $${P}_{t}-{P}_{xxt}+(\alpha +1)P{P}_{x}=\alpha {P}_{x}{P}_{xx}+P{P}_{xxx},$$

has been extensively studied by numerous researchers in^19–40^. Equation (2) simplifies to the CH equation when $\alpha =2$ takes the following form:

3 $${P}_{t}-{P}_{xxt}+3P{P}_{x}=2{P}_{x}{P}_{xx}+P{P}_{xxx}.$$

Equation (2) becomes the DP equation when $\alpha = 3$ takes the following form:

4 $${P}_{t}-{P}_{xxt}+4P{P}_{x}=3{P}_{x}{P}_{xx}+P{P}_{xxx}.$$

Both the CH and DP equations are bi-Hamiltonian and have isospectral problems associated with them^17^. The scattering and inverse scattering methods can be used to combine them in a formal way^16^. Such equations also allow peaked solutions, which are solitary waves often referred to as peakons. There are certain structural similarities between Eqs. (3) and (4), and they are necessarily dissimilar. Specifically, the isospectral problem of the DP equation is a third order problem, as compared to the second order problem of the CH equation^17^. Degasperis and Procesi^16^ have developed an equation of state by the technique of asymptotic integrability. Equation (2) is integrable only when α = 2 or α = 3. Equation (3) is associated with the case of $\alpha =2$ to represent shallow water waves that were originally derived as a set of approximations of the incompressible Euler equations. It was then demonstrated to be wholly integrable and to have a Lax pair formulation^16^. Similarly, Eq. (4) associated with $\alpha =3$ is also a modeller of shallow-water effects, and it has been shown to be integrable. The investigators have also shown that every equation in Eq. (2) favours single peakon solutions and multi-peakon structures^16^. The research is aimed at developing and improving the current level of research in the DP and CH frameworks in the following aspects:

5 $${P}_{t}-{P}_{xxt}+4{P}^{2}{P}_{x}=3{P}_{x}{P}_{xx}+P{P}_{xxx},$$

and

6 $${P}_{t}-{P}_{xxt}+3{P}^{2}{P}_{x}=2{P}_{x}{P}_{xx}+P{P}_{xxx},$$

respectively.

Equation (6) can be written as, according to Wazwaz^18^, in the following form:

7 $${P}_{t}-{P}_{xxt}+({P}^{3}{)}_{x}=\frac{1}{2}[({P}_{x}{)}^{2}{]}_{x}+(P{P}_{xx}{)}_{x}.$$

The scientific community has already been attracted by Eq. (7) because of the universal nature in modeling a wide variety of wave events. The formula depicts the worth of mathematicians, scholars, physicists, professionals, and engineers in such areas as image processing, fluid dynamics, and nonlinear optics. Simple and modified versions of the CH framework remain the basis for understanding the dynamics of waves, and numerous methods are to be considered. Camassa and Holm^15^ constructed the CH model to describe the propagation of the unidirectional shallow water waves with dispersive and nonlinear effects. It is also used in the computation of coastal regions, shallow waters, and harbor processes, and is usually employed in the long-term dynamics between peakons, a typical solitary wave solution, in the framework of ocean engineering^15^. In this framework, the revolution of smooth starting waveforms and waves breaking into breaking and persistent waves is realized successfully, and this defines the surface waves when there is non-hydrostatic pressure acting horizontally over a horizontal bed in shallow water^19^. The deformation experiment of integrable frameworks has received remarkable attention due to their ability to generate new and interesting mathematical frameworks. An example to be noticed is the deformation of the KdV framework, which introduces an augmentation to the integrable CH-NLS framework, derivative, within the framework of shallow water wave theory^20^. The results of deforming the NLS framework in a variant are also referred to as the CH-NLS framework^21,22^. Tian and Song^23^ remodeled the CH structure to introduce its nonlinearity. Boyd^24^ broadly studied the behavior of individual waves with slow growth in the traveling wave revolution. The results of Ali et al.^25^ provided accurate traveling wave solutions using the simplified MCH model, which provided wave constructions defined in terms of rational, exponential, hyperbolic, and trigonometric functions. Islam et al.^26^ analyzed the precise solutions of the SMCH equation and its wide applicability in mathematical physics and engineering.

There are many analytical processes that have examined the SMCH equation carried out by numerous physicists, mathematicians, and researchers^27–40^. Modifying the S-expansion technique of the SMCH equation, Hamida et al.^38^ obtained 31 different solutions expressed by hyperbolic, trigonometric, and exponential functions. Islam et al.^39^ analyzed the effect of wind and friction on the water waves under the SMCH model. Their bifurcation analysis found important equilibrium states that define the dynamics of the waves, and they have reported a few traveling solitons, such as kink, asymmetric, symmetric, as well as breather waves. The analysis indicates that the SMCH model is a useful tool because it can describe the actual wave behavior in the real world, particularly waves caused by wind, which provides practical data in the field of coastal engineering, energy, and shoreline protection. Akbulut et al.^40^ used an approach of a unified and better F-expansion technique to obtain exact traveling-wave solutions of the SMCH equation, which resulted in hyperbolic, trigonometric, and rational solutions. Their methodology incorporates several branches of solutions that are inaccessible to numerical methods, and is used in fluid dynamics, optics, and plasma physics. The simplicity and reliability of the methods are also supported by the 2D and 3D plots.

In the current study, the MCH framework is applied to find out the transformation of the physical nature of the solutions, as illustrated by the peakon explanations shown in Eq. (7), which involve lone waves and soliton constructions. The MG’/GE approach is an analytical and structured tool for describing NLPDEs, offering greater flexibility, an extensive solution area, less computational intricacy, and greater versatility for a variety of physical structures and physical limits in diverse systems and engineering applications. Despite its resources, the MG’/GE approach has many limitations, such as reliance on certain assumptions and parameter selections, limited applicability, and the inability to solve strongly nonlinear terms, as well as poor performance on numerical and high-dimensional problems. It best suits particular groups of NLEEs. The dynamical structure, equilibrium, and stability positions of the relevant 2D dynamical structure are also examined through an understanding of bifurcation theory. Bifurcations and chaotic behaviours were also examined using phase-plane analysis and showed considerable qualitative differences between the dynamical structures in question. This work has enhanced our knowledge of complex nonlinear processes in the study of quasiperiodic and chaotic wave phenomena due to external influences. Our examination will be implemented using the MG’/GE approach^41^.

### Existing gaps in this work

A close examination of the past research on the MCH model indicates the presence of some significant gaps. So far, the MG’/GE approach has not been used on this model. Also, linear stability analysis of the model and its solutions has not been explored. Also, more powerful chaos characterization methods, such as strange attractors, recurrence plots, bifurcation diagrams, and fractal dimension analysis, have not been applied in previous studies. These gaps provide evidence of the necessity to investigate the MCH model more thoroughly and systematically. To fill these gaps, the current study will use the MG’/GE approach to find the exact analytical solutions of the MCH equation. In addition to the derivation of solutions, the work has performed a stability analysis of the model in detail. Nonlinear and chaotic dynamics of the system are also explored with the help of such advanced instruments as strange attractors, recurrence plots, bifurcation diagrams, and fractal dimension analysis. The research offers new information on the MCH model to the best of the knowledge of the authors because it combines these methodologies, thus providing a more comprehensive view of the analytical and dynamical characteristics of this model.

### Motivation, comparative novelty, and key contributions of the present study

The MCH equation has a significant role in modeling nonlinear dispersive wave phenomena as it occurs in the nonlinear dynamics of shallow water systems, nonlinear fluid mechanics, and other nonlinear physical systems. Despite the broad analytical tools available, including the sine–cosine method, tanh method, and other classical expansion methods that have been used to analyze NLEEs, it is difficult to obtain a variety of exact solutions of the MCH equation that are structurally rich in nature. Most of the currently available methods are constrained by either accuracy, flexibility, or their capacity to resolve several forms of traveling-wave structures. This is the gap that drives the creation of more resilient and flexible tools of analysis, which can generate a wider category of precise solutions with a definite physical connection. To overcome these shortcomings, the current research proposes a refined analytical model based on the MG’/GE approach.

Contrary to the past studies where most of the techniques, including the sine cosine method^18^ and the tanh method^18^, had been used to obtain approximate solutions of the modified Camassa Holm (MCH) model, the current work has the MG’/GE approach^41^ to obtain the exact solutions of the MCH model. There is also a symbolic bifurcation analysis, which increases the interpretability of the solutions and gives a more profound idea of the processes within a system. Previous studies on the MCH model have concentrated solely on solitary wave solutions. Conversely, we demonstrate that these earlier reported results are recoverable as special cases in the larger solution structure obtained using the MG’/GE approach. The effect of physical parameters on solitary solutions is thoroughly examined, and the results are consistent with the amplitude and width of the solution, which agrees with previous research. Another major contribution of this work is the study of quasi-periodicity and chaotic behavior of the perturbed system. Examining such nonlinear phenomena, the research demonstrates the efficacy of the MCH model in describing the complicated processes of wave propagation and the transmission of signals. The capability to consider nonlinear and chaotic dynamics gives the model a new understanding of the emergence of chaotic behavior in nonlinear systems, presenting the possibility of application in the production of predictive models, diagnostic devices, and control mechanisms to stabilize complex wave and signal systems.

Its novelty is in the fact that the systematic use of the MG’/GE approach to obtain a large family of traveling-wave solutions of the MCH equation has been carried out, in trigonometric, hyperbolic, and rational forms. More to the point, the approach is able to create new and varied wave structures, including single and multiple bright and dark solitons, multiple singular solitons, which, so far as we know, have not been reported concurrently with the MCH framework using a single analytical structure. The technique is also characterized by high computational efficiency, better accuracy, and high adaptability, especially when extended to fractional-order nonlinear models. The core findings of the research can be outlined as follows:

(i) Formulation of the MG’/GE approach that could generate a rich assortment of precise closed form traveling-wave solutions of the MCH equation.
(ii) Production of several new and structurally rich waveforms, such as bright, dark, singular, multiple, and rational solitons, and indicating the flexibility of the method.
(iii) Detailed graphical representation of the behavior of the solutions via the 3D plot, contour plot, density plot, 2D plot of time evolution, as well as 3D revolving image in order to show the dynamics of the MCH waves.
(iv) Generalization of the approach to the MCH framework, which would emphasize its strength and its capacity to apply to more general nonlinear physical systems.
(v) To develop the MG’/GE approach as a more accurate and versatile method than current methods of analysis, like sine–cosine and tanh methods.

In general, this study has provided an analytical scheme that is mathematically rigorous and physically meaningful for the NLEEs. The findings not only provide a better insight into the traveling-wave phenomena of nonlinear dispersive systems but also provide a solid foundation for future advancement in fluid and plasma physics, the propagation of optical waves, and other fields of applied sciences.

### Advantages and limitations of the MG′/GE method

The MG’/GE approach is an effective and systematic approach to finding the precise soliton solutions of NLEEs. This can be used to convert complex partial differential equations (PDEs) into simpler ordinary differential equations (ODEs) that are easier to analyze analytically by a wave transformation, combined with an auxiliary equation that is a Riccati equation. The ability of the MG’/GE approach to produce a broad range of soliton solutions is another strength of the approach. With the parameters of the solution structure, the method is able to provide hyperbolic, trigonometric, and rational forms, such as W-shaped solitons, bright and dark periodic single static solitons, bright and dark bell solitons, and periodic rogue wave solitons. This enables modeling the nonlinear propagation of waves in the system, which is of fractional order, in detail. Moreover, the MG’/GE approach gives a unique solution, offering a single framework of discrete, periodic, and mixed-type wave solutions, as opposed to others that are often limited to a particular form or set of conditions. It allows for the calculation of soliton properties to an accurate level, including amplitude, phase velocity, and dispersion relation.

Jacobian elliptic functions are used to enable a smooth change of hyperbolic and trigonometric functions, which makes convergence faster and minimizes numerical errors. The method can also correct boundary conditions and nonlinear coefficients, making stable solutions available for a large variety of initial conditions. As the MG’/GE approach is characterized by numerous benefits, it has certain limitations. The main one is that it greatly depends on choosing a proper transformation of the wave and balancing the higher-order and nonlinear terms of derivatives. This is especially challenging with very complicated or higher-order nonlinear systems. In addition, the approach is mostly tailored to making soliton solutions, which can overlook other important dynamic phenomena, including blow-up behaviors, multi-soliton interactions, or non-traveling wave structures. Thus, the MG’/GE approach may be very useful in studying solitons but is somewhat limited in its usage in investigating the entire dynamics of multifaceted systems.

### Organization of this research

This study is structured as follows. In Section “Introduction”, the background and motivation of the study, a thorough literature review, the formulation of the governing MCH model, a description of the existing research gaps, and the innovative contributions of the given work are outlined. This section also includes the motivation, originality, and contributions subsection, which aims to explain the importance of the current study. Section “The modified-expansion method” explains the analysis techniques used, focusing on the MG’/GE approach. Section “Solving the modified CH equation via the modified-expansion method” outlines the solution plan and shows how the suggested schemes would be applied in the MCH equation. Section “Results and discussion” gives a detailed insight into the results obtained, a comparison with current methods, and offers numerous graphical representations. Section “Quasi-periodicity and chaos analysis after the introduction of different perturbation terms” performs a stability analysis of the model with the linear stability theory and the use of the Hamilton tool to test the validity of the obtained solutions. Section “Stability analysis of the obtained solutions” explores the chaos of the system using advanced measures of quality. Lastly, Section “Conclusion and future work” concludes with a brief summary of the key findings, limitations, and possible future research directions.

## The modified $({G}{\prime}/G)$-expansion method

We consider the equation below:

8 $$W(P,\frac{\partial P}{\partial t},\frac{\partial P}{\partial x},\frac{{\partial }^{2}P}{{\partial }^{2}x},\frac{{\partial }^{2}P}{\partial t\partial x},\frac{{\partial }^{2}P}{{\partial }^{2}t},\cdots \cdots )=0.$$

Initially, employ the travelling variable:

9 $$P(\xi )=P(x,t),\xi =x-ct.$$

Using Eq. (9) in Eq. (8), we get:

10 $$F(P,P {^{\prime}},P {^{\prime}}{^{\prime}},\cdots \cdots )=0.$$

Again, we consider the ansatz equation:

11 $$P\left(\xi \right)={\sum }_{i=-M}^{M}{E}_{i}\{N(\xi ){\}}^{i},$$

where $\left( \xi \right) = \left( {\frac{{G^{\prime } \left( \xi \right)}}{G\left( \xi \right)} + \frac{\lambda }{2}} \right)^{i}$, $\left| {E_{ - M} } \right| + \left| {E_{M} } \right| \ne 0$ and $G\left(\xi \right)$ satisfies the equation

12 $${G}^{^{\prime\prime} }(\xi )+\lambda {G}^{{^{\prime}}}(\xi )+\mu G(\xi )=0$$

where ${E}_{i}(\pm 1,\pm 2,....,\pm M)$, λ and μ are constant coefficients. The homogeneous balancing method can be used to find the positive integer M in Eq. (11). Equation (12) reveals that,

13 $$N(\xi )=T-N(\xi {)}^{2},$$

where $T=\frac{{\lambda }^{2}-4\mu }{4}$ and T is calculated by λ and μ. So, $N(\xi )$ satisfies Eq. (13), which produces:

$$N\left( \xi \right) = \left\{ {\begin{array}{*{20}l} {\sqrt T \tan h(\sqrt T \xi ),T > 0;} \hfill \\ {\sqrt T \cot h(\sqrt T \xi ),T > 0;} \hfill \\ {\frac{1}{\xi }T = 0;} \hfill \\ { - \sqrt { - T} \tan (\sqrt { - T} \xi ),T < 0;} \hfill \\ { - \sqrt { - T} \cot (\sqrt { - T} \xi ),T < 0.} \hfill \\ \end{array} } \right.$$

The phases of the MG’/GE approach are outlined in an algorithm that we present. The process for applying the approach to NLEEs of the following algorithm is clearly and methodically summarized by this visual aid:

Step 1: Start with the given PDE: $F(u,{u}_{t},{u}_{x},{u}_{xx},\dots )=0$

Step 2: Apply the traveling wave transformation $u\left(x,t\right)=u\left(\xi \right), where$ $\xi =x-vt$

Step 3: Convert ODE.

Step 4: Taking the series expansion of the ODE.

Step 5: Balance the terms and solve for the unknown coefficients.

Step 6: Substitute the constants into the sub-equation and construct $u(\xi )$

Step 7: obtained solution.

## Solving the modified CH equation via the modified $(G{\prime}/G)$ -expansion method

The following equation is created by utilizing the change of variables $P(x,t)=P(\xi ),$ $\xi =x-ct$ with Eq. (7):

14 $$-cP+cP {^{\prime}} {^{\prime}}+{P}^{3}-PP {^{\prime}} {^{\prime}}-0.5(P{^{\prime}}{)}^{2}=0.$$

where the prime notation denotes the derivative concerning the variable $\xi$, balances the highest order nonlinear and linear terms present in Eq. (14). We have attained $M=2$. Consequently, it is found that:

15 $$P(\xi )={E}_{-2}{N}^{-2}(\xi )+{E}_{-1}{N}^{-1}(\xi )+{E}_{0}{N}^{0}(\xi )+{E}_{1}{N}^{1}(\xi )+{E}_{2}{N}^{2}(\xi ).$$

Then, by setting the coefficients of this polynomial equal to zero, a system of linear algebraic equations is formed, and the set is obtained as its solution:

Phase-I: $$c=1,$$ $$\mu =0.25{\lambda }^{2}+0.25,$$ $${E}_{0}=1,$$ $${E}_{1}=0,$$ $${E}_{2}=0,$$ $${E}_{-1}=0$$ and $${E}_{-2}=0.5.$$

Phase-II: $c=2,$ $\mu =0.25{\lambda }^{2}-0.25,$ ${E}_{0}=-2,$ ${E}_{1}=0,$ ${E}_{2}=0,$ ${E}_{-1}=0$ and ${E}_{-2}=0.5.$

Phase-III: $c=1,$ $\mu =0.25{\lambda }^{2}+0.25,$ ${E}_{0}=1,$ ${E}_{1}=0,$ ${E}_{2}=8,$ ${E}_{-1}=0$ and ${E}_{-2}=0.$

Phase-IV: $c=2,$ $\mu =0.25{\lambda }^{2}-0.25,$ ${E}_{0}=-2,$ ${E}_{1}=0, {E}_{2}=8,$ ${E}_{-1}=0$ and ${E}_{-2}=0.$

Phase-V: $$c=1,$$ $$\mu =0.25{\lambda }^{2}+0.0625,$$ $${E}_{0}=0,$$ $${E}_{1}=0,$$ $${E}_{2}=8,$$ $${E}_{-1}=0$$ and $${E}_{-2}=0.03125.$$

Phase-VI: $c=1,$ $\mu =0.25{\lambda }^{2}-0.25,$ ${E}_{0}=-1,$ ${E}_{1}=0,$ ${E}_{2}=8,$ ${E}_{-1}=0$ and ${E}_{-2}=0.03125.$

By replacing the aforementioned Phase-I values in Eq. (15), we obtain

$${P}_{11}(\xi )=1+0.5\times \{\sqrt{T}\mathit{tan}h(\sqrt{T}\xi ){\}}^{-2},T>0.$$

$${P}_{12}(\xi )=1+0.5\times \{\sqrt{T}\mathit{cot}h(\sqrt{T}\xi ){\}}^{-2},T>0.$$

$${P}_{13}(\xi )=1+0.5\times \{\frac{1}{\xi }){\}}^{-2},T=0.$$

$${P}_{14}(\xi )=1+0.5\times \{-\sqrt{-T}\mathit{tan}(\sqrt{-T}\xi ){\}}^{-2},T<0.$$

$${P}_{15}(\xi )=1+0.5\times \{-\sqrt{-T}\mathit{cot}(\sqrt{-T}\xi ){\}}^{-2},T<0.$$

By replacing the aforementioned Phase-II values in Eq. (15), we obtain

$${P}_{21}(\xi )=-2+0.5\times \{\sqrt{T}\mathit{tan}h(\sqrt{T}\xi ){\}}^{-2},T>0.$$

$${P}_{22}(\xi )=-2+0.5\times \{\sqrt{T}\mathit{cot}h(\sqrt{T}\xi ){\}}^{-2},T>0.$$

$${P}_{23}(\xi )=-2+0.5\times \{\frac{1}{\xi }){\}}^{-2},T=0.$$

$${P}_{24}(\xi )=-2+0.5\times \{-\sqrt{-T}\mathit{tan}(\sqrt{-T}\xi ){\}}^{-2},T<0.$$

$${P}_{25}(\xi )=-2+0.5\times \{-\sqrt{-T}\mathit{cot}(\sqrt{-T}\xi ){\}}^{-2},T<0.$$

By replacing the aforementioned Phase-III values in Eq. (15), we obtain

$${P}_{31}(\xi )=1+8\times \{\sqrt{T}\mathit{tan}h(\sqrt{T}\xi ){\}}^{2},T>0.$$

$${P}_{32}(\xi )=1+8\times \{\sqrt{T}\mathit{cot}h(\sqrt{T}\xi ){\}}^{2},T>0.$$

$${P}_{33}(\xi )=1+8\times \{\frac{1}{\xi }){\}}^{2},T=0.$$

$${P}_{34}(\xi )=1+8\times \{-\sqrt{-T}\mathit{tan}(\sqrt{-T}\xi ){\}}^{2},T<0.$$

$${P}_{35}(\xi )=1+8\times \{-\sqrt{-T}\mathit{cot}(\sqrt{-T}\xi ){\}}^{2},T<0.$$

By replacing the aforementioned Phase-IV values in Eq. (15), we obtain

$${P}_{41}(\xi )=-2+8\times \{\sqrt{T}\mathit{tan}h(\sqrt{T}\xi ){\}}^{2},T>0.$$

$${P}_{42}(\xi )=-2+8\times \{\sqrt{T}\mathit{cot}h(\sqrt{T}\xi ){\}}^{2},T>0.$$

$${P}_{43}(\xi )=-2+8\times \{\frac{1}{\xi }){\}}^{2},T=0.$$

$${P}_{44}(\xi )=-2+8\times \{-\sqrt{-T}\mathit{tan}(\sqrt{-T}\xi ){\}}^{2},T<0.$$

$${P}_{45}(\xi )=-2+8\times \{-\sqrt{-T}\mathit{cot}(\sqrt{-T}\xi ){\}}^{2},T<0.$$

By replacing the aforementioned Phase-V values in Eq. (15), we obtain

$${P}_{51}(\xi )=8\times \{\sqrt{T}\mathit{tan}h(\sqrt{T}\xi ){\}}^{2}+0.03125\times \{\sqrt{T}\mathit{tan}h(\sqrt{T}\xi ){\}}^{-2},T>0.$$

$${P}_{52}(\xi )=8\times \{\sqrt{T}\mathit{cot}h(\sqrt{T}\xi ){\}}^{2}+0.03125\times \{\sqrt{T}\mathit{cot}h(\sqrt{T}\xi ){\}}^{-2},T>0.$$

$${P}_{53}(\xi )=8\times \{\frac{1}{\xi }){\}}^{2}+0.03125\times \{\frac{1}{\xi }){\}}^{-2},T=0.$$

$${P}_{54}(\xi )=8\times \{-\sqrt{-T}\mathit{tan}(\sqrt{-T}\xi ){\}}^{2}+0.03125\times \{-\sqrt{-T}\mathit{tan}(\sqrt{-T}\xi ){\}}^{-2},T<0.$$

$${P}_{55}(\xi )=8\times \{-\sqrt{-T}\mathit{cot}(\sqrt{-T}\xi ){\}}^{2}+0.03125\times \{-\sqrt{-T}\mathit{cot}(\sqrt{-T}\xi ){\}}^{-2},T<0.$$

By replacing the aforementioned Phase-VI values in Eq. (15), we obtain

$${P}_{61}(\xi )=-1+8\times \{\sqrt{T}\mathit{tan}h(\sqrt{T}\xi ){\}}^{2}+0.03125\times \{\sqrt{T}\mathit{tan}h(\sqrt{T}\xi ){\}}^{-2},T>0.$$

$${P}_{62}(\xi )=-1+8\times \{\sqrt{T}\mathit{cot}h(\sqrt{T}\xi ){\}}^{2}+0.03125\times \{\sqrt{T}\mathit{cot}h(\sqrt{T}\xi ){\}}^{-2},T>0.$$

$${P}_{63}(\xi )=-1+8\times \{\frac{1}{\xi }){\}}^{2}+0.03125\times \{\frac{1}{\xi }){\}}^{-2},T=0.$$

$${P}_{64}(\xi )=-1+8\times \{-\sqrt{-T}\mathit{tan}(\sqrt{-T}\xi ){\}}^{2}+0.03125\times \{-\sqrt{-T}\mathit{tan}(\sqrt{-T}\xi ){\}}^{-2},T<0.$$

$${P}_{65}(\xi )=-1+8\times \{-\sqrt{-T}\mathit{cot}(\sqrt{-T}\xi ){\}}^{2}+0.03125\times \{-\sqrt{-T}\mathit{cot}(\sqrt{-T}\xi ){\}}^{-2},T<0.$$

## Results and discussion

This section provides an extensive comparison of current methods of analysis and numbers with the help of graphic illustrations of the solutions obtained.

Although modified and simplified CH equations have been studied by many researchers^15–40^, Wazwaz was the only researcher to discuss the MCH equation with the sine–cosine and tanh techniques. Most of the previous studies have been based on analysis or numerical simulation to obtain solutions of the MCH equation, but they have not entirely explored the dynamic and chaotic nature of the equation. Conversely, the current work proposes and implements the MG’/GE approach to the MCH equation, using other auxiliary equations to obtain a wider range of solutions, some of which have never been published before. The outcomes of this technique are unique and extend beyond those of prior research. Furthermore, chaotic dynamics of the model are explored in a systematic manner through the bifurcation diagrams, strange attractors, Lyapunov exponents, and other techniques, and give a complete picture of the nonlinear behavior of the system. The MG’/GE approach allows a great number of nonlinear evolution equations due to its efficiency and flexibility, which implies a high probability of being used in further studies. It is evident that the additional solutions obtained in this study are novel and original, as evidenced by comparing the current results with the ones in the literature, as summarized in Table 1.

**Table 1:** Compared to previously reported results.

Research	Method used	Solutions	Solution type	Bifurcation analysis	Chaotic analysis	Quasi-periodicity
The present study	the modified $\left({G}^{{^{\prime}}}/G\right)$-expansion method	Thirty solutions	Trigonometric, rational, and hyperbolic solutions: single singular, double singular, multiple bright, multiple dark, multiple singular, and singular solitons			
Wazwaz^13^	The sine–cosine method	Two solutions	Hyperbolic solutions			
Wazwaz^13^	The tanh method	Two solutions	Hyperbolic solutions			

To use graphical representations effectively, you need a thorough comprehension of various kinds of plots, such as revolving 3D plots, 3D surface plots, contour maps, density (or heat) maps, and 2D time-evolution graphs. Although they help to make complicated behaviours easier to understand, represent, and examine, these graphical tools are crucial when investigating NLEEs, particularly when it comes to soliton solutions. This section highlights the importance of each graph type in soliton analysis. Our method is more accurate and reliable, as we compare our results with those obtained from the same equations using existing methods. The visualisations show the solutions to the transformed CH framework under different parameter settings.

The structure, symmetry, and changing behaviour of wave events are best captured through visual representations, such as mesh or wireframe displays, revolving 3D graphs, surface plots, and contour plots. These charts usually show the evolution of solutions to NLEEs using three axes, which typically stand for spatial coordinates, amplitude, and time. When examining systems with multiple variables, 3D graphs are especially important because they allow researchers to visualize how solutions change across two spatial dimensions and a third variable, such as strength or time. For example, contour plots render it easier to observe the geographic and temporal fluctuation of wave strength by showing lines of constant value. Density plots, frequently referred to as heat maps, are useful for identifying regions with peak intensities, troughs, or singular behaviours since they use colour to illustrate magnitude at each point. By revolving around an axis, rotating 3D plots provide a thorough overview and efficiently display the proportions and geometric features of soliton formations.

Since they maintain their shape while flowing at speeds in accordance, solitons are especially fascinating in nonlinear wave examinations. Researchers may gain a deeper understanding of how solitons originate and interact in various chemical products through the use of these visual instruments. 2D time-evolution graphs, on the other hand, are helpful to observe the evolution of wave features over time, providing data on motion, interaction, and stability. Besides being essential to understanding intricate behaviours like soliton collisions and wave blow-ups, these graphic representations also help a larger audience understand what has been discovered. They may demonstrate patterns that are difficult to determine through mathematical analysis alone, and they help validate both analytical and numerical decisions. These techniques increase our overall understanding of nonlinear structures and are thoroughly applicable in domains ranging from fluid dynamics to plasma physics.

**Fig. 1:**
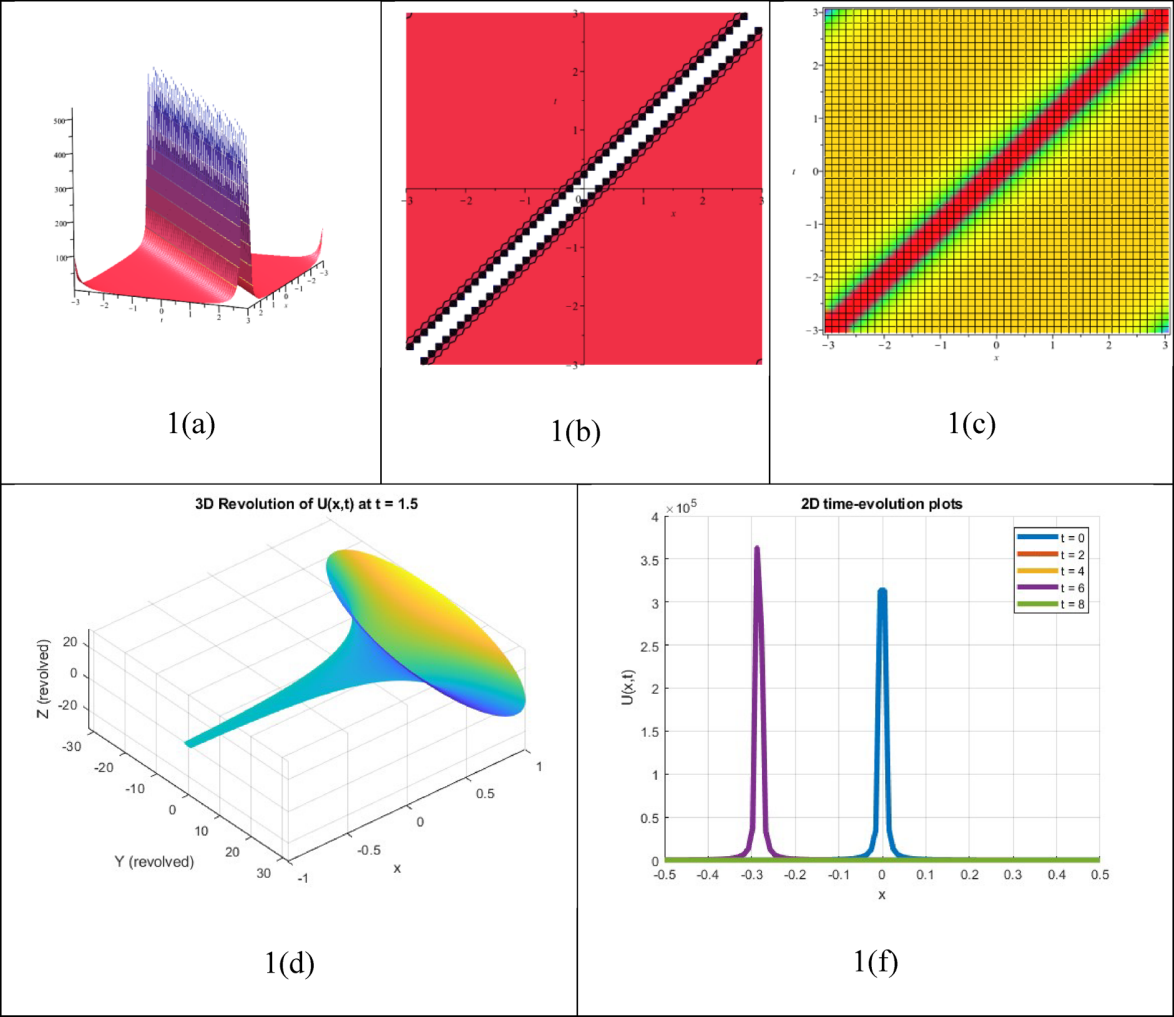
3D, Contour, density, 3D revolving and 2D time-evolution profiles of the solution ${P}_{14}(\xi )$.

A single waveform response of an NLEE that retains its shape while moving but exhibits a sudden spike (blow-up) at a specific time or space can be recognized as a unique soliton solution. Such an approach can be expressed as

16 $$P\left( {x,t} \right) = \frac{A}{{(x - ct)^{n\prime } }}$$

where A represents the amplitude, c denotes the wave speed, and n > 0 controls the strength of the singularity. The attained answer ${P}_{14}(\xi )$ shares related properties with Eq. (16) and is therefore identified as a single singular soliton answer, demonstrated in Fig. 1. The corresponding three-dimensional, Contour, density, 3D revolving, and 2D time-evolution profiles of the solution ${P}_{14}(\xi )$ are also demonstrated in Fig. 1. Despite not being constantly physically feasible, these solutions give insight into extreme behaviours throughout nonlinear structures and may be employed to model waveforms that demonstrate infinite energy or steepness at an exact location. These approaches are typically idealized concepts in fields like fluid dynamics, optics, or plasma physics.

**Fig. 2:**
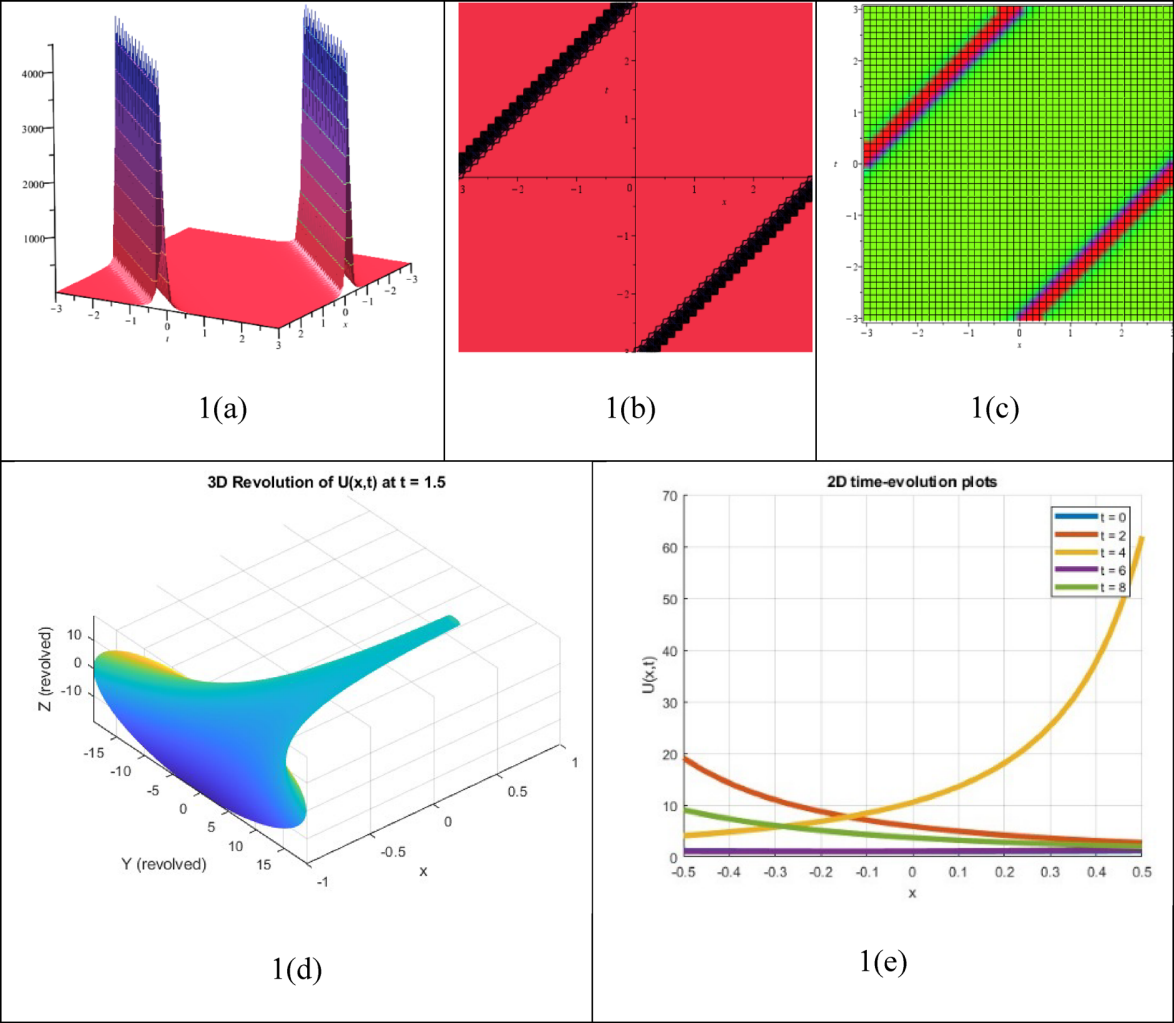
3D, Contour, density, 3D revolving and 2D time-evolution profiles of the solution ${P}_{15}(\xi )$.

A double singular soliton solution is a type of NLEEs that explains how two solitary waves interact, with each wave creating a singularity that leads to very high or infinite amplitude at certain points in time or space. The resulting answer ${P}_{15}(\xi )$, which was determined as a double singular soliton solution, is shown in Fig. 2. The graph also displays the subsequent 2D time evolution, 3D contour, density, and 3D revolving characteristics of the solution ${P}_{15}(\xi )$. These solutions are important for understanding phenomena such as wave blow-up, nonlinear superposition, and energy concentration in various fields, including fluid dynamics, mathematical physics, nonlinear optics, and plasma physics. However, they are often theoretical and not always possible in real life due to their unique characteristics.

**Fig. 3:**
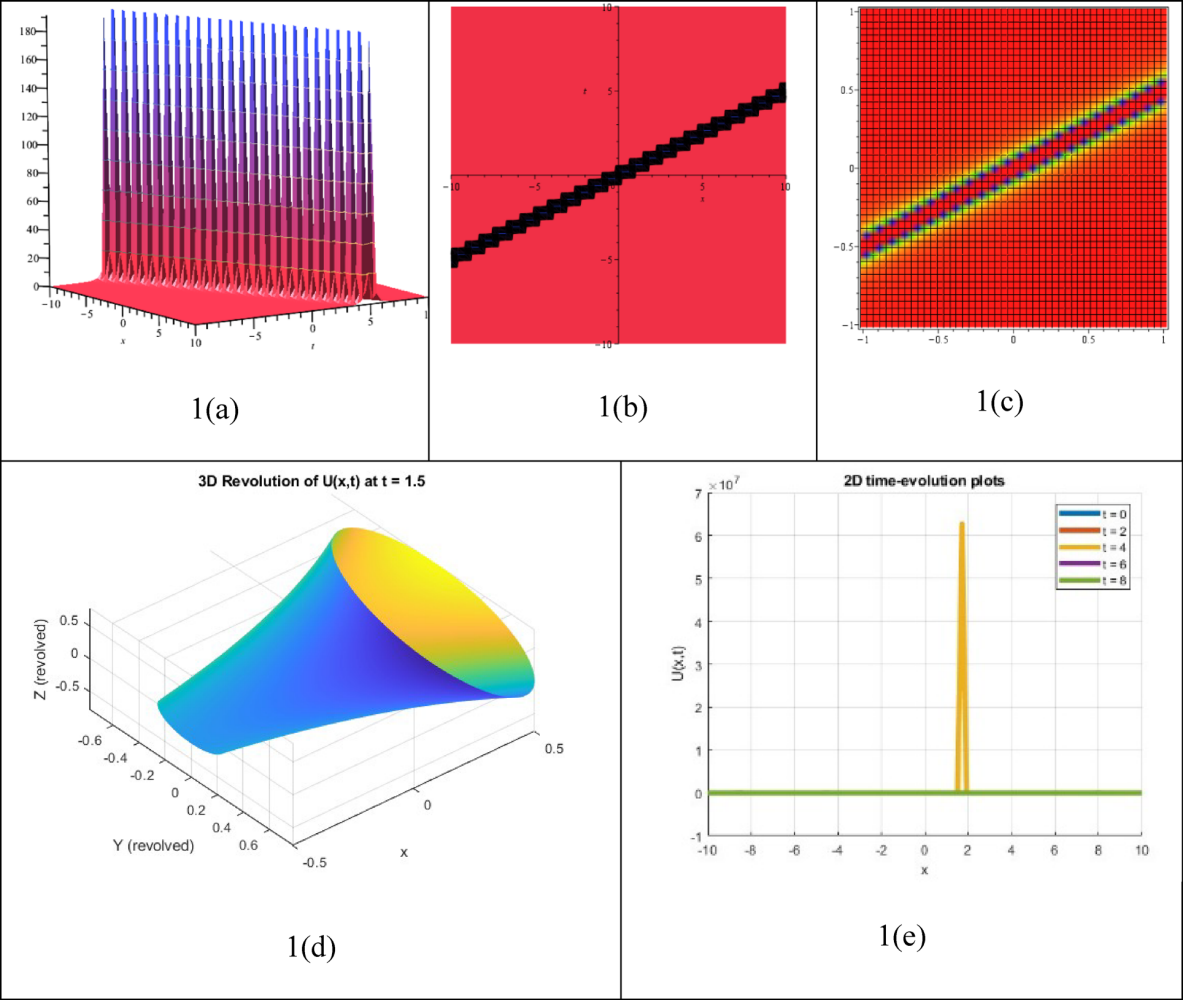
3D, Contour, density, 3D revolving and 2D time-evolution profiles of the solution ${P}_{21}(\xi )$.

The presence or coexistence of two or more bright solitons, which are waveforms with sharp peaks and no background that maintain their shape and strength while travelling through a nonlinear medium, can be identified by a multiple bright soliton solution, showing a specific reaction to an NLEE. The outcome of the solution ${P}_{21}(\xi )$, which has been identified as a multiple outstanding soliton solution, has been shown in Fig. 3. The associated 3D contour, density, 3D revolving, and 2D time-evolution spectra of the solution ${P}_{21}(\xi )$ can be observed in the picture. Diverse bright soliton solutions must be developed to appreciate nonlinear wave behavior in various categories, especially plasma physics, water wave dynamics, optical fiber communications, and Bose–Einstein condensates.

**Fig. 4:**
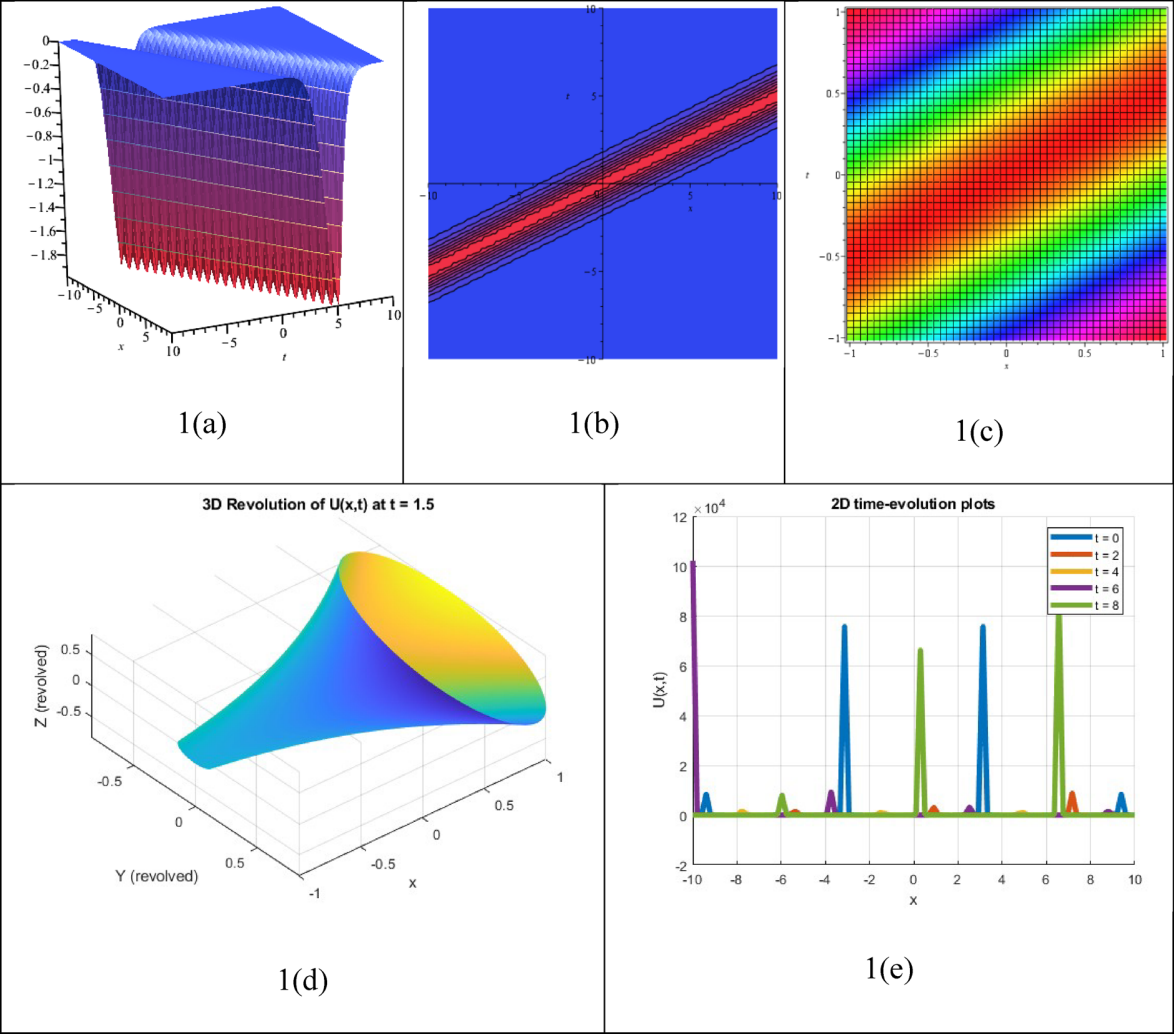
3D, Contour, density, 3D revolving and 2D time-evolution profiles of the solution ${P}_{22}(\xi )$.

A multiple dark soliton solution, an accurate result of an NLEE, stands for the relationship or simultaneous involvement of more than one dark soliton, which has localized waves with apparent decreases or intensity troughs in an infinite nonzero surrounding that preserve their shape and speed while traveling through a nonlinear medium. The corresponding solution ${P}_{22}(\xi )$, which has been recognized as a multiple dark soliton solution, appears in Fig. 4. The figure presents the corresponding 3D, contour, density, 3D revolving, and 2D time-evolution profiles of the solution ${P}_{22}(\xi )$. Numerous dark soliton solutions are useful in various fields, including fluid dynamics, Bose–Einstein condensates, optical fiber signal processing and transmission, and plasma physics.

**Fig. 5:**
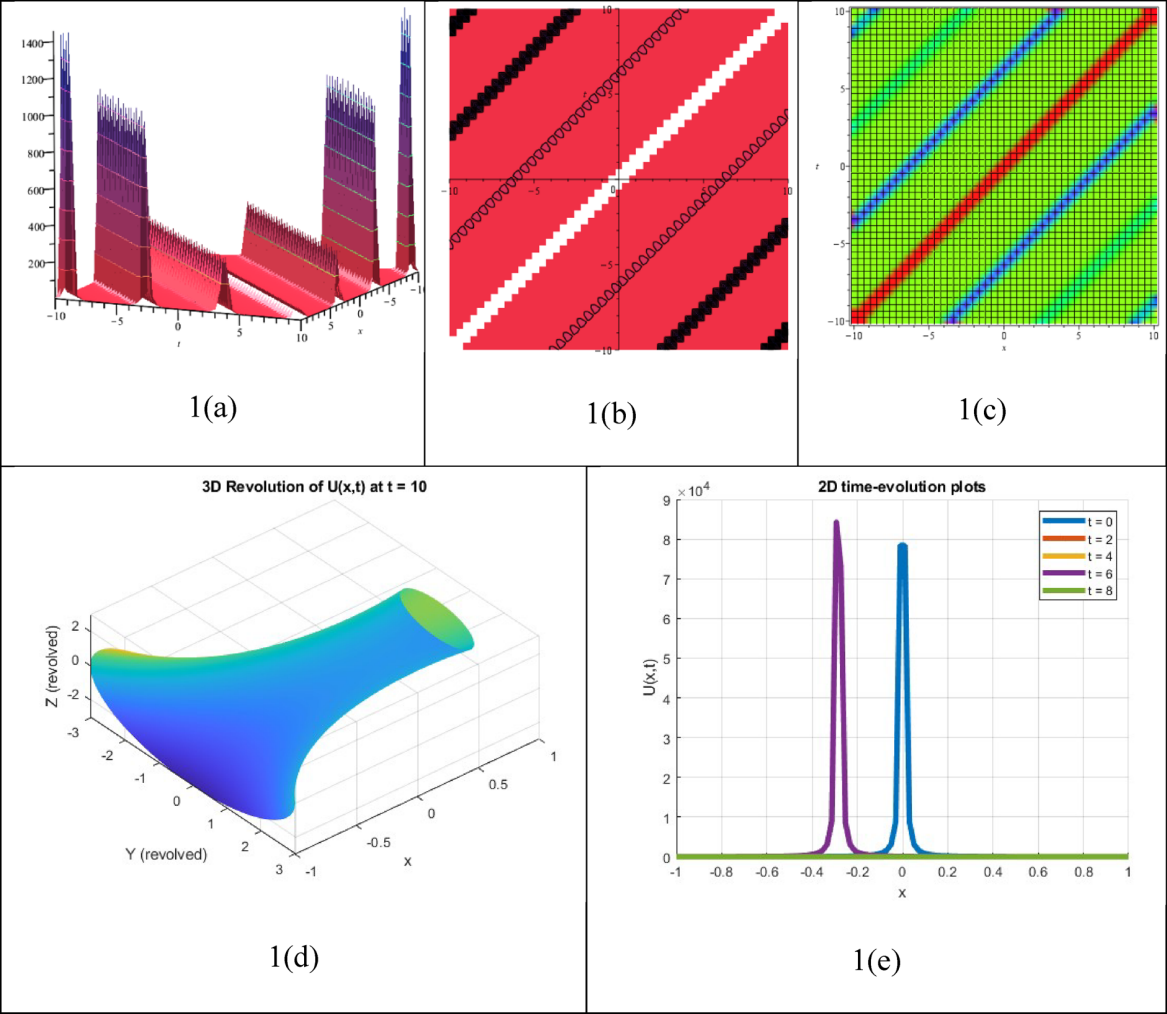
3D, Contour, density, 3D revolving and 2D time-evolution profiles of the solution ${P}_{54}(\xi )$.

The interaction or simultaneous presence of two or more singular solitons—solitary waves with singularities like infinite amplitude or blow-up at particular locations in space or time—while preserving their distinct wave characteristics during propagation is described by a multiple singular soliton solution, which is a precise solution to an NLEE. Figure 5 shows the obtained solution ${P}_{54}(\xi )$, which was determined to be a multiple singular soliton solution. The figure also presents the corresponding 3D, contour, density, 3D revolving, and 2D time-evolution profiles of the solution ${P}_{54}(\xi )$.

**Fig. 6:**
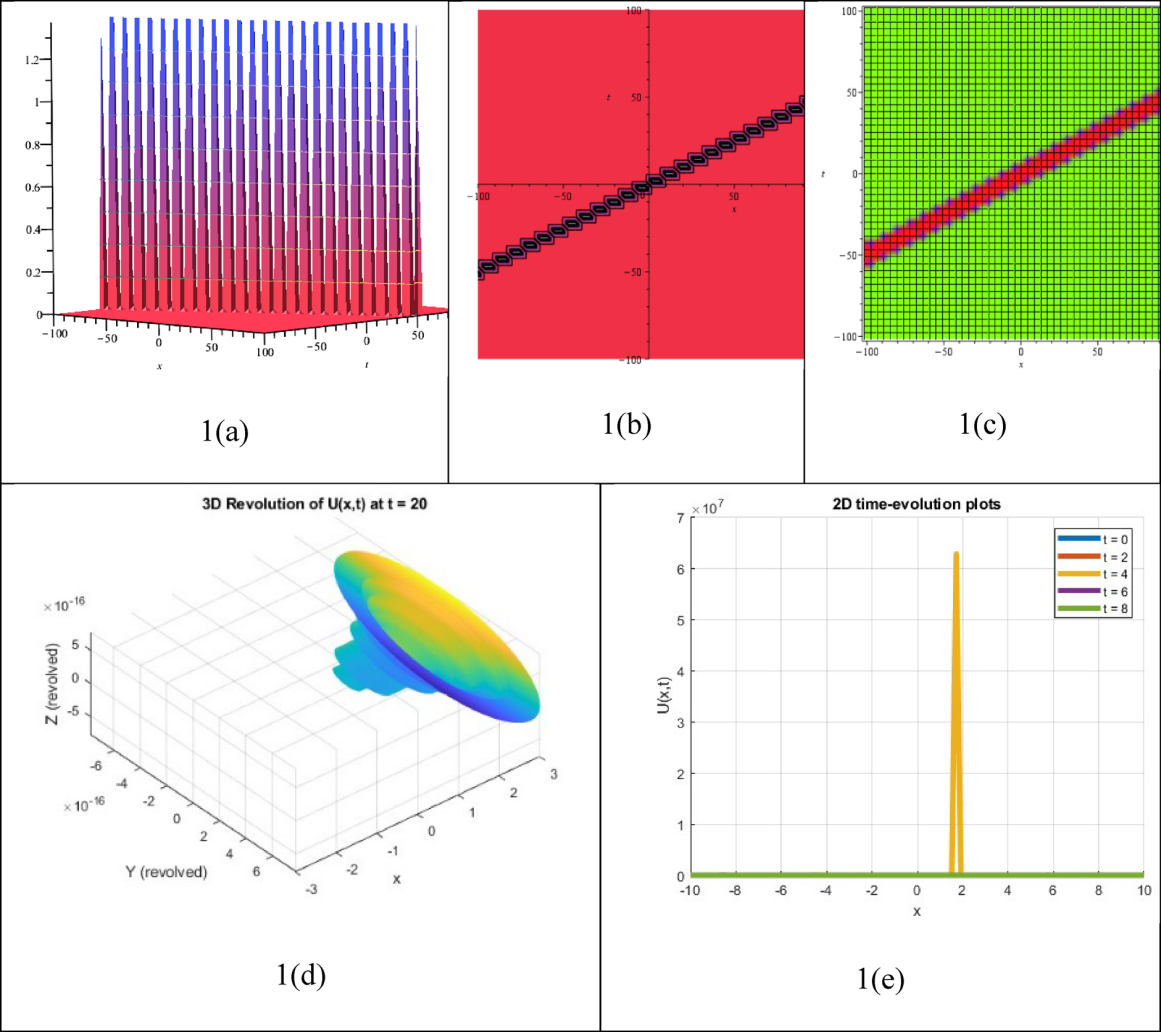
3D, Contour, density, 3D revolving and 2D time-evolution profiles of the solution ${P}_{61}(\xi )$.

The obtained solution ${P}_{61}(\xi )$ presents in Fig. 6, which was determined to be a double singular soliton solution. The figure displays the corresponding 3D, contour, density, 3D revolving, and 2D time-evolution profiles of the solution ${P}_{61}(\xi )$.

**Fig. 7:**
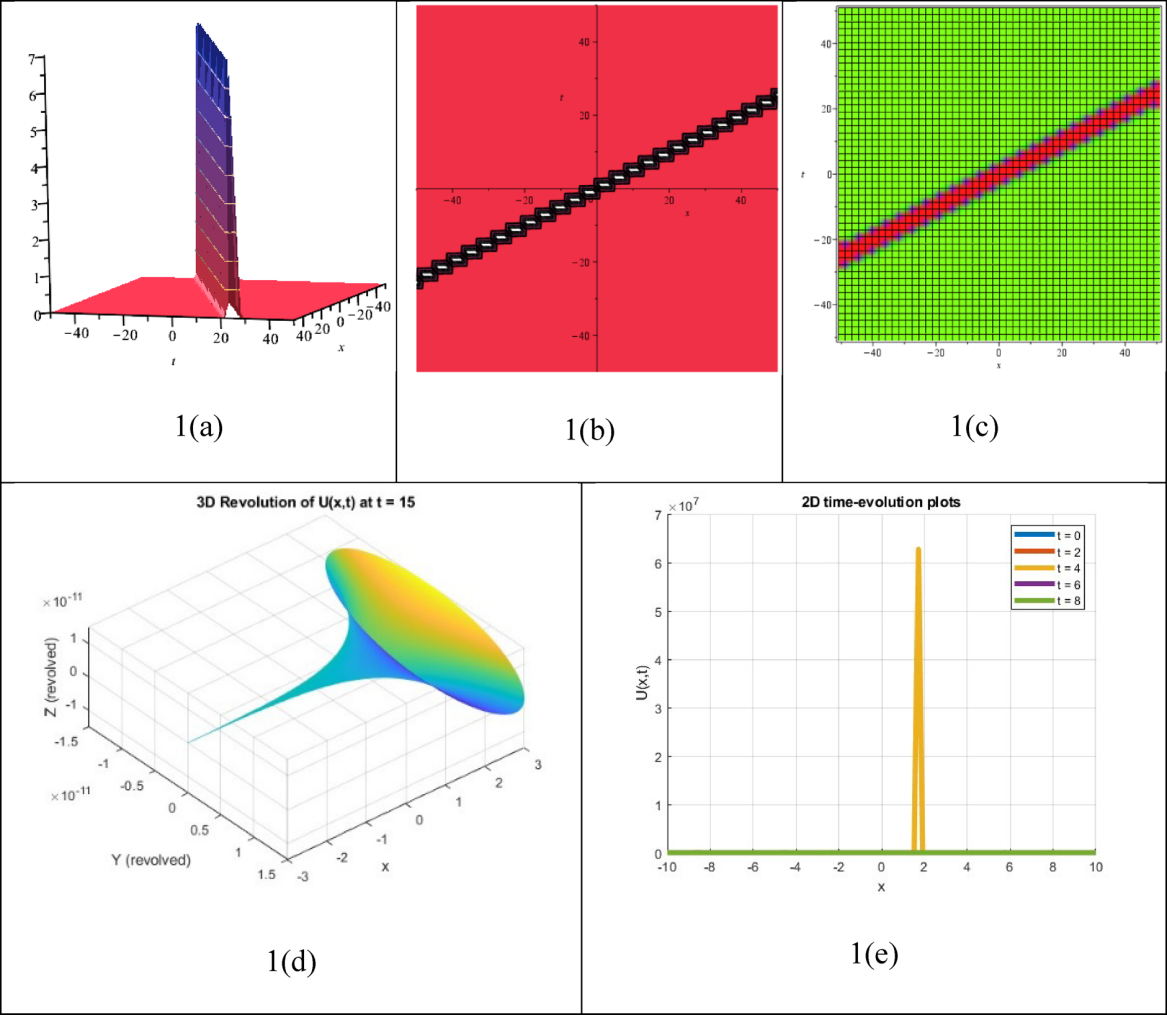
3D, Contour, density, 3D revolving and 2D time-evolution profiles of the solution ${P}_{62}(\xi )$.

One type of solution to an NLEE that describes a single wave with a singularity (e.g., infinite amplitude or steep gradient) at a particular point in space or time that nevertheless maintains soliton-like properties (e.g., shape and stability during propagation) is called a singular soliton solution. Figure 7 illustrates the obtained solution ${P}_{62}(\xi )$, which was determined to be a singular soliton solution. It presents the corresponding 3D, contour, density, 3D revolving, and 2D time-evolution profiles of the solution ${P}_{62}(\xi )$.

## Bifurcation analysis of dynamical systems

Bifurcation is defined as a qualitative shift in behavior in a system due to a small change in one parameter^42^. Analyzing this, the Galilean transformation is applied to Eq. (9) and its dynamic structure is derived. Bifurcation analysis involves the reduction of the nonlinear equation of higher order into a first-order equation $\frac{dP(\xi )}{d\xi }=F(x, L)$, where x presents the state-vector, L denotes a bifurcation parameter, and F signifies a smooth vector field and the linearization, Jacobian analysis, and strict stability analysis of a smooth vector field. This formulation is first-order and is the basis of phase portraits, bifurcation diagrams, and the nonlinear dynamics of incompressible fluid surfaces. Equation (14) can be converted into several steps for bifurcation analysis as follows:

*Step 1*: Conversion to First-Order System: Let: $X = P$ and $Y = P{^{\prime}}$, then:

17 $$\left\{ {\begin{array}{*{20}c} {X^{\prime } = Y } \\ {Y^{\prime} = \frac{{\left[ {cX{ } - { }X^{3} { } + { }0.5{ }Y^{2} } \right]}}{{\left( {c{ } - { }X} \right)}}, } \\ \end{array} } \right.\;\;\;{\mathrm{Assuming}}\;\;c{ } \ne { }X.$$

*Step 2*: Finding Fixed Points: Set $X{^{\prime}} = 0$ and $Y{^{\prime}} = 0$. From $X{^{\prime}} = Y = 0$. From ${Y}^{{^{\prime}}}=\frac{\left[cX - {X}^{3}\right]}{\left(c - X\right)} = 0\Rightarrow cX - {X}^{3}= 0 \Rightarrow X(c - {X}^{2}) = 0$ $\Rightarrow X = 0$ or $X = \pm \sqrt{c}.$ So, the fixed points are: $(0, 0), (\surd c, 0), (-\surd c, 0)$.

*Step 3*: Jacobian Matrix: Let ${f}_{1} = Y$, and ${f}_{2} =\frac{\left[cX - {X}^{3} + 0.5 {Y}^{2}\right]}{\left(c - X\right)}$. Then the Jacobian matrix is:

$$J=\left[\begin{array}{cc}\frac{\partial {f}_{1}}{\partial X}& \frac{\partial {f}_{1}}{\partial Y}\\ \frac{\partial {f}_{2}}{\partial X}& \frac{\partial {f}_{2}}{\partial Y}\end{array}\right]$$. Here, $\frac{\partial {f}_{1}}{\partial X}=0$ and $\frac{\partial {f}_{1}}{\partial Y}=1.$ Then, $\frac{\partial {f}_{2}}{\partial X}=\frac{-3{X}^{2}+ c}{c - X} +\frac{-{X}^{3}+ cX + 0.5{Y}^{2}}{{\left(c - X\right)}^{2}}$ and $\frac{\partial {f}_{2}}{\partial Y}=\frac{Y}{c-X}$. Therefore, the Jacobian matrix will be $$J=\left[\begin{array}{cc}0& 1\\ \frac{-3{X}^{2}+ c}{c - X} +\frac{-{X}^{3}+ cX + 0.5{Y}^{2}}{{\left(c - X\right)}^{2}}& \frac{Y}{c-X}\end{array}\right]$$.

## At a fixed point $(0, 0)$:

Jacobian at $(0, 0)$: $${J}_{\left(\mathrm{0,0}\right)}=\left[\begin{array}{cc}0& 1\\ 1& 0\end{array}\right] .$$ i.e. Eigenvalues $\lambda =\pm 1$, hence at $\left(\mathrm{0,0}\right),$ we got a saddle point (unstable).

## At a fixed point $(\surd {\boldsymbol{c}},0)$:

Jacobian at $(\surd c, 0)$: $${J}_{\left(\sqrt{c},0\right)}=\left[\begin{array}{cc}0& 1\\ -\frac{2\mathrm{c}}{\text{c }- \sqrt{\mathrm{c}}}& 0\end{array}\right]$$, i.e. Eigenvalues $\lambda = \pm \sqrt{\frac{2c}{\left(\sqrt{c}- c\right)}}$.

 → Saddle Point (real eigenvalues of opposite sign if ${\boldsymbol{s}}\in (0,1)$).

 → Center (pure imaginary eigenvalues if ${\boldsymbol{s}}>1$).

## At a fixed point $(-\surd {\boldsymbol{c}},0)$:

**Fig. 8:**
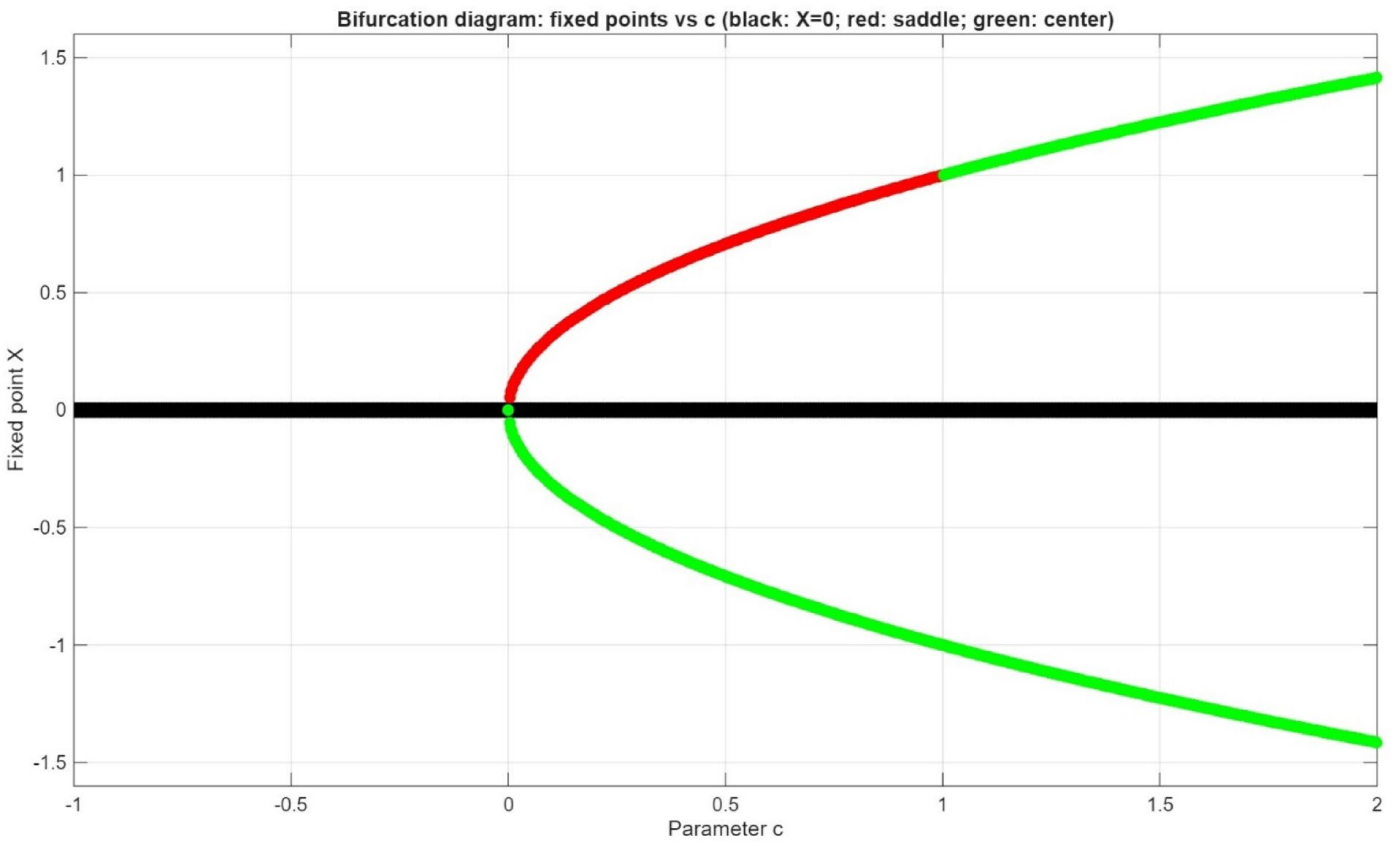
At $c=0$ the system bifurcates and appears to have two more fixed points as $c>0.$

Jacobian at $(-\surd c, 0)$: $${J}_{\left(-\sqrt{c},0\right)}=\left[\begin{array}{cc}0& 1\\ -\frac{2\mathrm{c}}{\mathrm{c}+ \sqrt{\mathrm{c}}}& 0\end{array}\right]$$, i.e., Eigenvalues: $\lambda = \pm \sqrt{\frac{-2c}{\left(\sqrt{c}+ c\right)}}$, Pure imaginary, hence we get a center (Fig. 8).

**Fig. 9:**
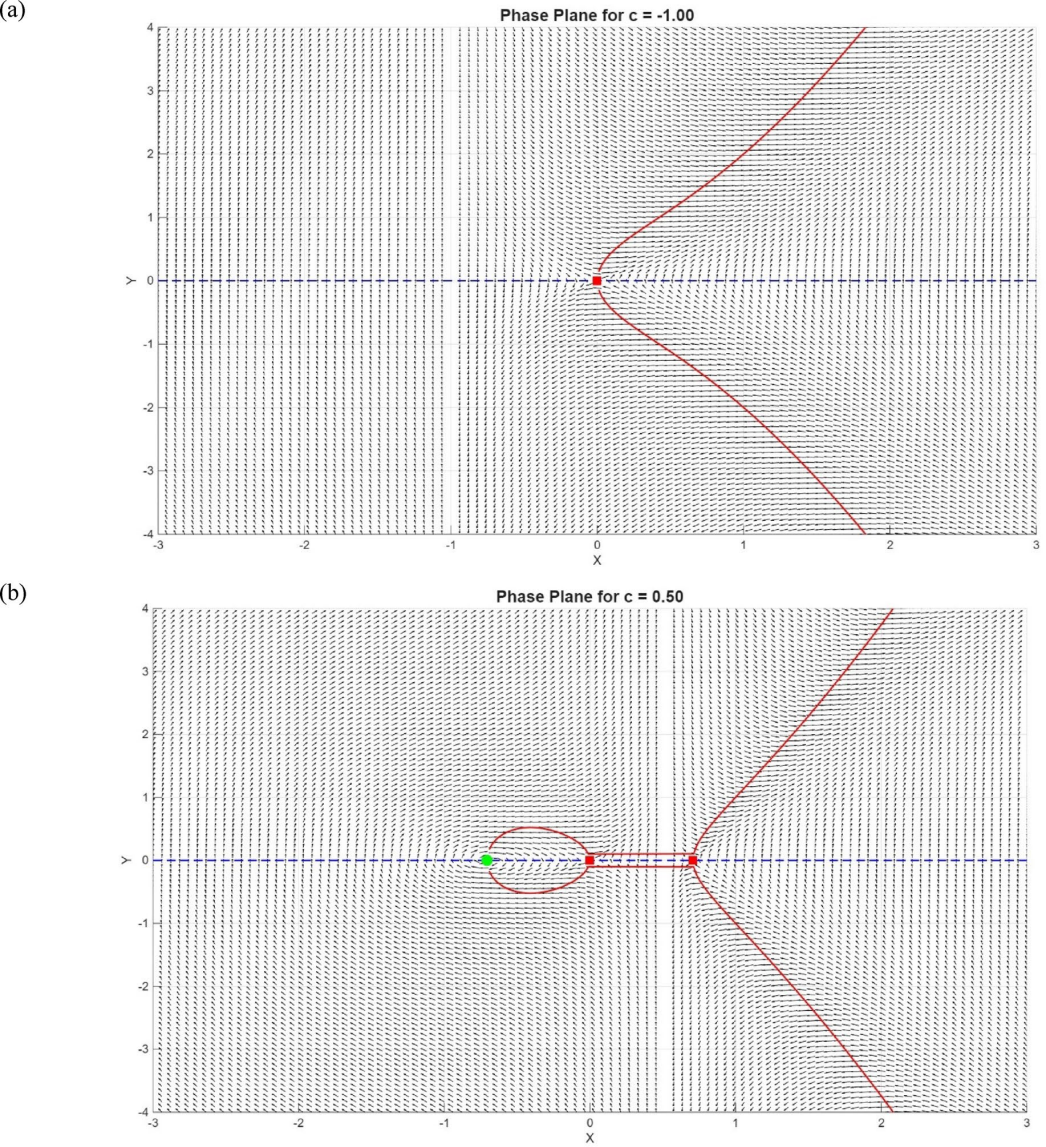

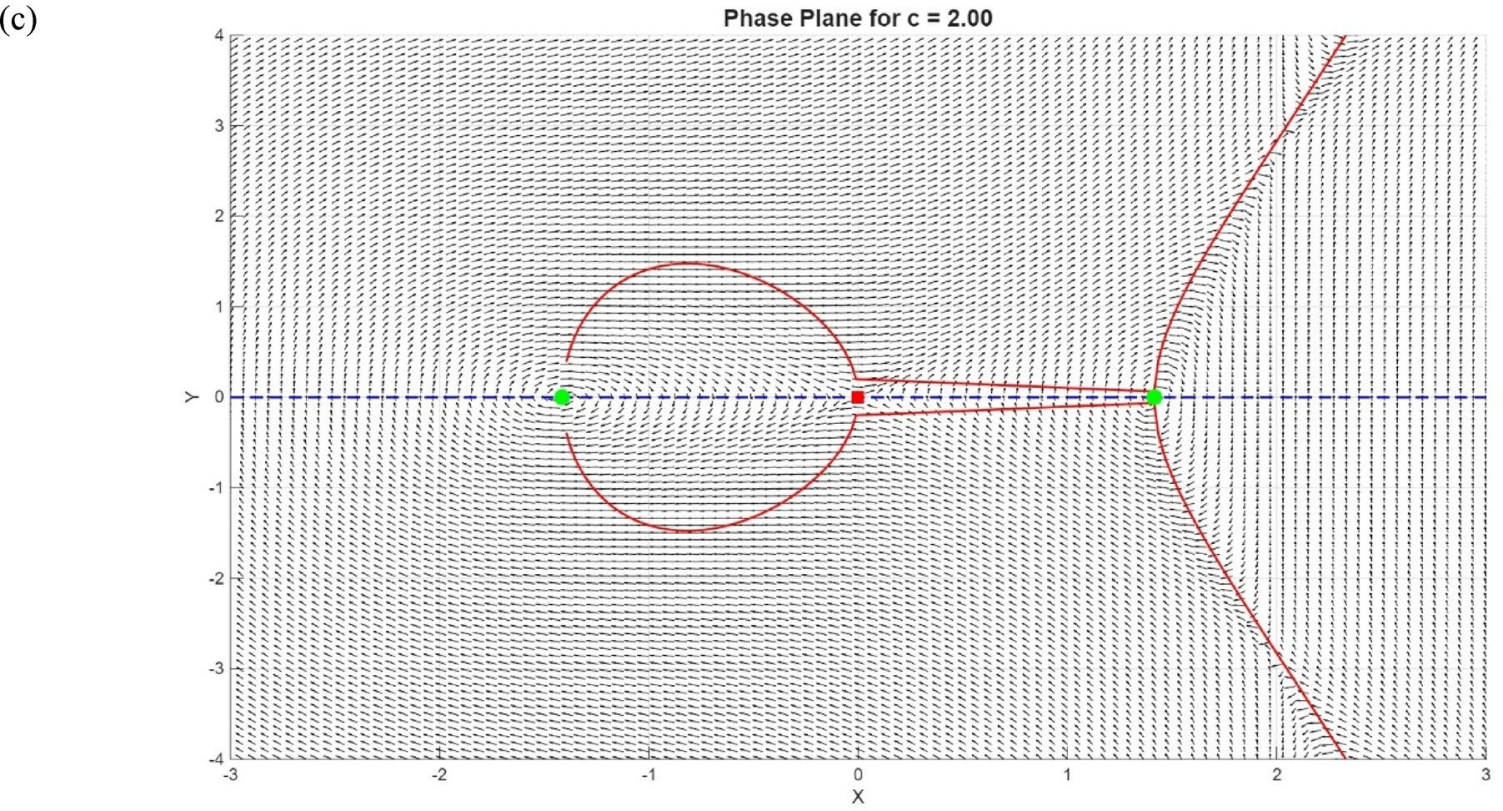
2D Phase portrait of the system with $c=-1, c=0.5$ and $c=2$.

*Case 1*: If $c<0$, our system has only one fixed point at $(\mathrm{0,0})$ which is a saddle point in Fig. 9a.

*Case 2*: If $0<c<1$, our system has three fixed points $(-\sqrt{c},0), (\mathrm{0,0})$ and $(\sqrt{c},0)$ which are centered, saddle, and saddle, respectively, in Fig. 9b.

*Case 3*: If $c>1,$ the present system has three fixed points $(-\sqrt{c},0), (\mathrm{0,0})$ and $(\sqrt{c},0)$ but which are centered, saddle, and centered, respectively, in Fig. 9c.

At $c=0,$ the system bifurcates and appears to have two more fixed points as $c>0.$ A bifurcation diagram of the equilibrium points c versus dissipation of fixed points changes as shown in Fig. 8.

**Fig. 10:**
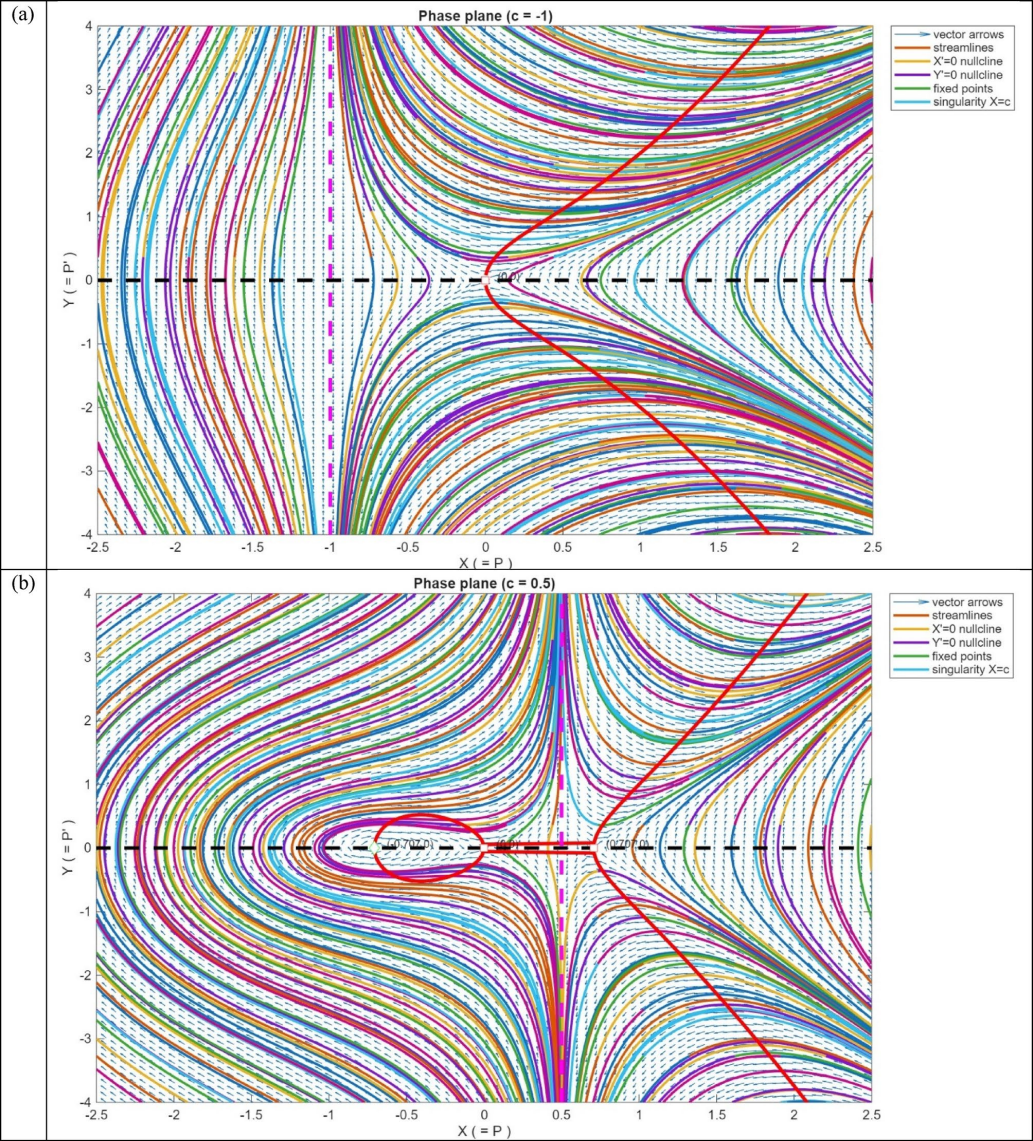

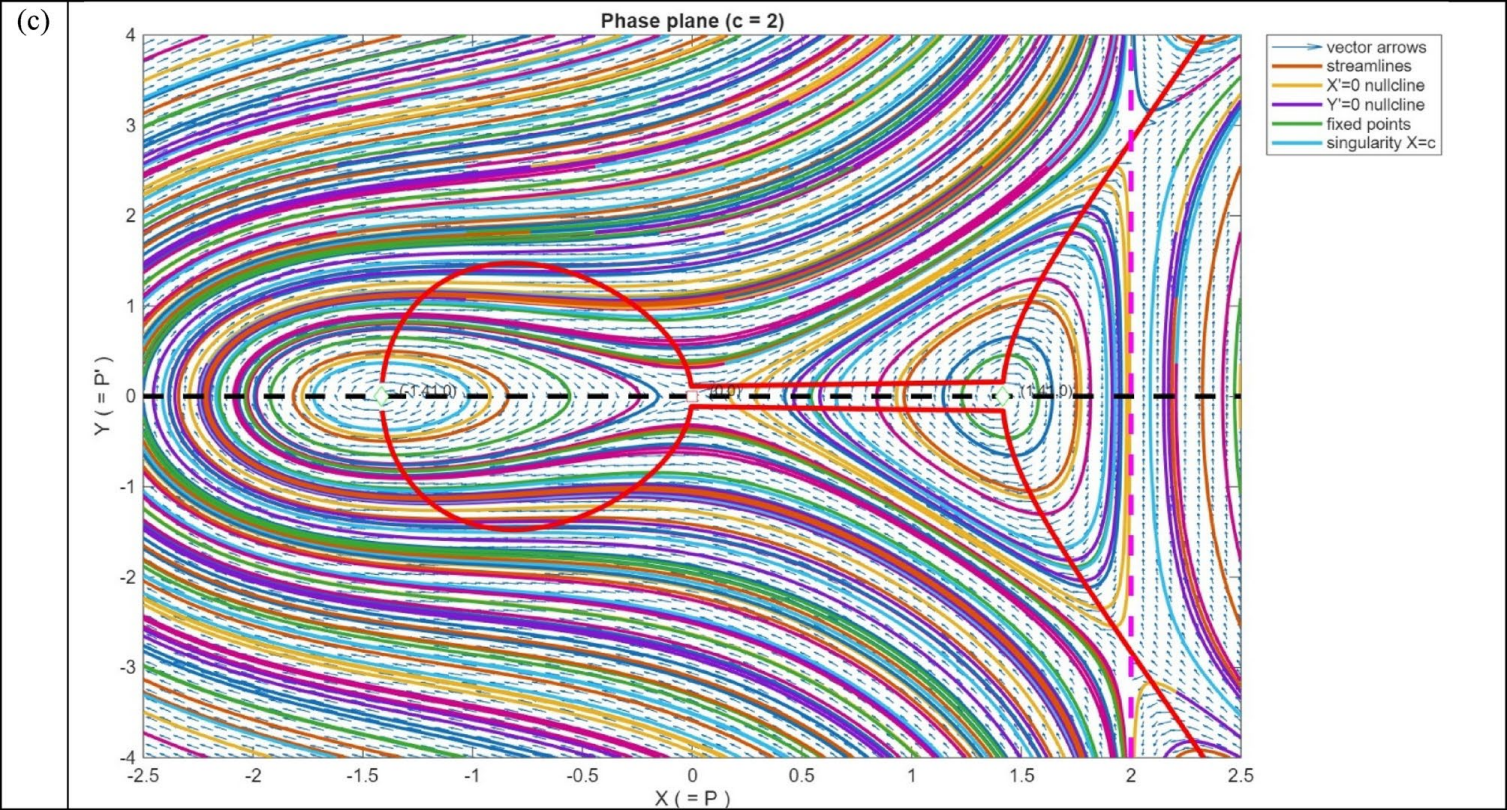
Phase-plane analysis of the system with streamlines, vector field, fixed points, nullclines, and singular line.

The behaviour of the system in the phase plane is depicted in Fig. 10. The vector field is displayed as arrows indicating the direction and the strength of the flow. Figure 10a shows the streams, which are the system trajectories with time. The nullclines are indicated by dashed lines: the black dashed line is the $Y=0$, and the red dashed line is the ${Y}^{{^{\prime}}}=0$. These lines indicate the positions where the respective derivatives disappear, which are used to find fixed points. The fixed points are marked with hollow markers, whose style reflects the linear stability of the fixed points: red squares denote saddles (unstable) and the green diamonds denote centers (neutrally stable) in Fig. 10b. The system has the birthing of $\pm \sqrt{c}$ at $c=0$ which is a classic pitchfork bifurcation. $X=c$ indicated x-axis magenta dashed vertical line indicates a singularity in the X-Axis vector field. Trajectories must not cross this line; the flow can blow up around in Fig. 10c. The denominator c-X vanishes at c = X, and this makes the vector field singular at c = 1. Practically, these values should not be plotted, and near the singular line, the trajectories may blow up, or the numerical integration may become erratic.

In Fig. 10, the black dashed line represents $Y=0$ nullcline, the red dashed line signifies ${Y}^{{^{\prime}}}=0$ nullcline. Hollow markers indicate fixed points: red squares indicate saddles (unstable), green diamonds indicate centers (neutrally stable). The $X=c$ indicates that the magenta dashed vertical line in the vector field causes a singularity in the field, along which the trajectories escape by blowing up. At $c=0$, a pitchfork bifurcation, in which the equilibria of $\pm \sqrt{c}$ are the same, and this shows the symmetry breaking in the system.

**Fig. 11:**
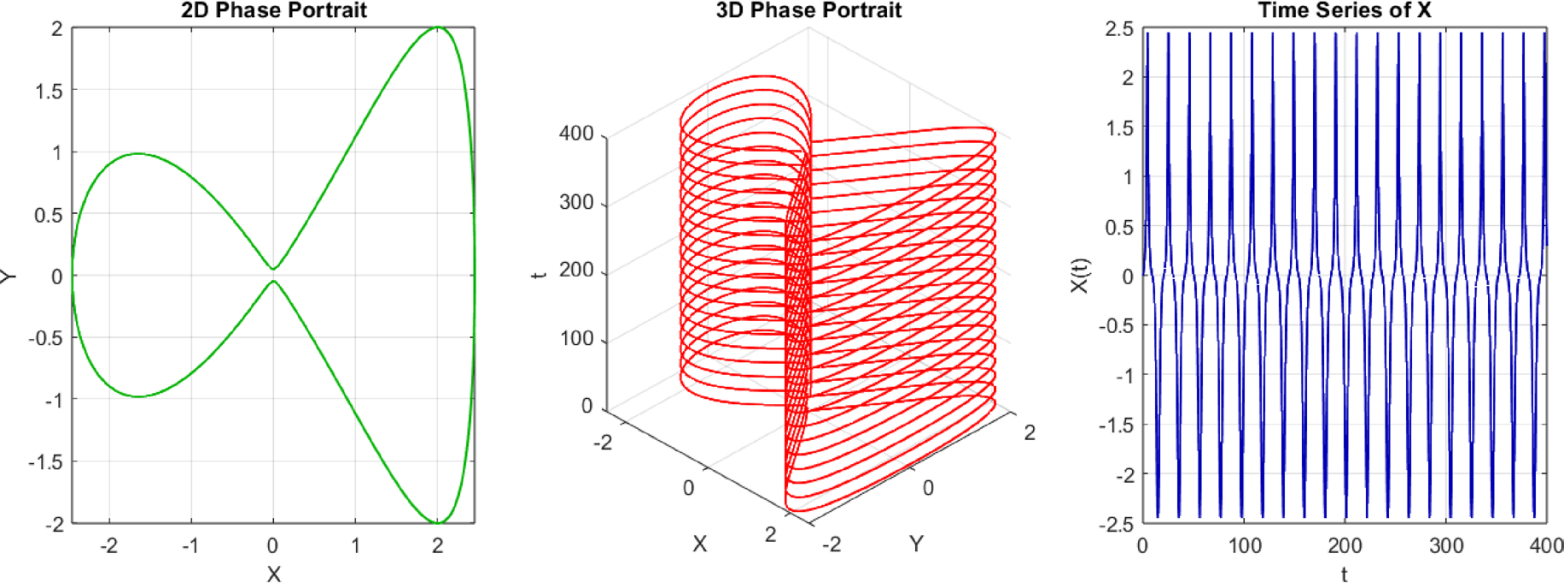
2D, 3D phases and time series of the trajectory at $s=3$, starting at the initial point IC = [0, 0.05]. The time series figure indicates the regularity of the trend, thereby favoring a non-chaotic system.

The 2D phase portrait indicates the plot of the system pathway in the $(X, Y)$ plane with $s=3$ and initial condition IC = [0, 0.05]. The path is smooth and closed or bounded, stating that the movement is regular and predictable. Physically, this represents that the system is developing in a stable way about a point of fixation or a limit cycle, without any indication of chaotic dispersion, as shown in Fig. 11a. The phase diagram visualization is extended to the 3D case (usually in time or another system variable) by a 3D phase diagram, which provides a more comprehensive view of the curve in the phase space. The trajectory is a smooth, non-intersecting curve in three dimensions, indicating the absence of chaotic behavior and the presence of structured, quasi-periodic, or periodic motion, as illustrated in Fig. 11b. The time series plot indicates the process of time dependence of the system variables based on the initial condition. The regularity and stability of the system are evidenced through smoothness and repetition, which proves that the system is non-chaotic at $s=3$. This means, in a physical sense, that the response of the system can be predicted in the long run, and one is not subjected to jumps or extreme sensitivity to initial conditions, as depicted in Fig. 11c.

## Quasi-periodicity and chaos analysis after the introduction of different perturbation terms

The present section examines the chaotic behavior of the next shifting system^43^ by considering perturbed terms. This study discusses phase portraits of two and three dimensions. The current study examines the transition between chaotic and quasi-periodic dynamics of the system introduced by the introduction of various perturbations, as listed in the table that follows^44^, based on three sources. In Table 2, some common characteristics of periodic, quasi-periodic, and chaotic dynamics are evident in the time series, phase portraits, and Poincare map.

**Table 2:** System behavior characterized using various representations.

Behavior	Time series	Phase portrait	Poincare map
Periodic	Repeats Exactly	Closed loop	One or a few points
Quasi-periodic	Smooth, Layered Cycles	Dense loops on a torus	Closed smooth curve
Chaotic	Irregular and Unpredictable	Strange attractors	Scattered points

Consider some perturbation terms and observe the behavior of the perturbed system.

18 $$X^{\prime } = Y\;{\mathrm{and}}\;Y^{\prime } = \frac{{\left[ {cX{ } - { }X^{3} { } + { }0.5{ }Y^{2} } \right]}}{{\left( {c{ } - { }X} \right)}} + P\left( t \right)$$

### Perturbation term of the trigonometric cos function

Let $P(t) =A cos bt$, then Eq. (18) becomes

19 $$X^{\prime } = Y\;{\mathrm{and}}\;Y^{\prime } = \frac{{\left[ {cX{ } - { }X^{3} { } + { }0.5{ }Y^{2} } \right]}}{{\left( {c{ } - { }X} \right)}} + A\cos bt.$$

**Fig. 12:**
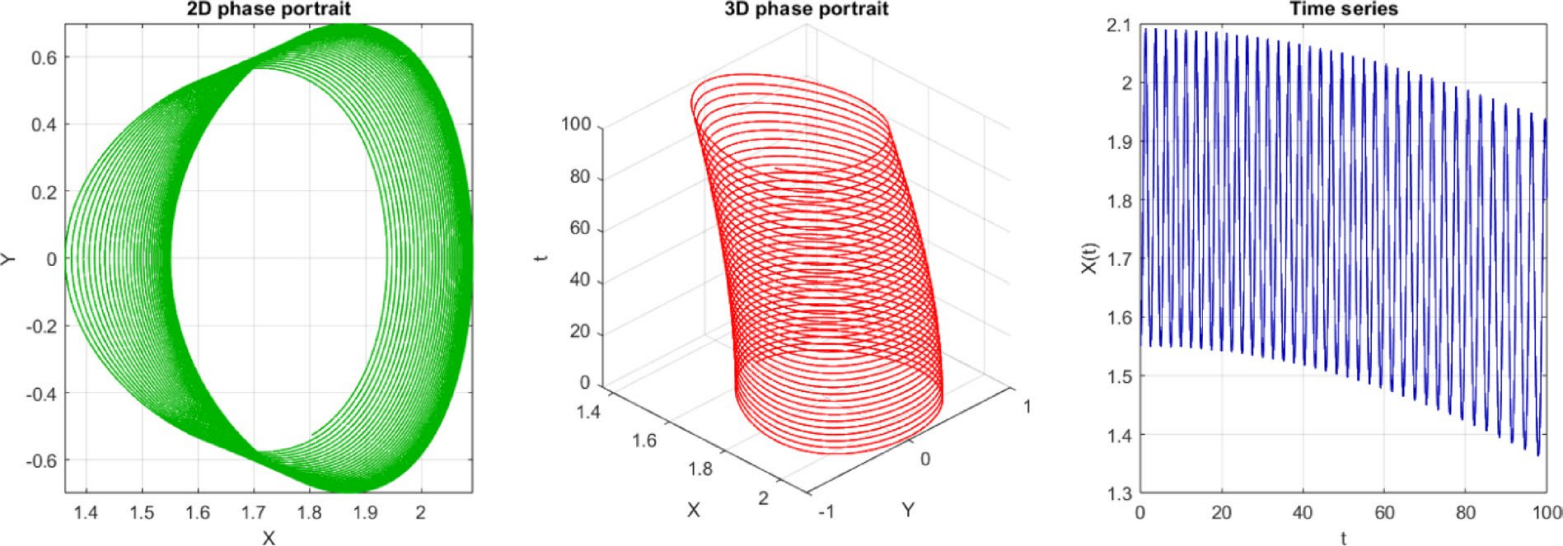
2D, 3D phase portrait and time series of the perturbed system at $C=3, A=0.6 b=0.02$ with IC $=[\mathrm{1.55,0}]$.

**Fig. 13:**
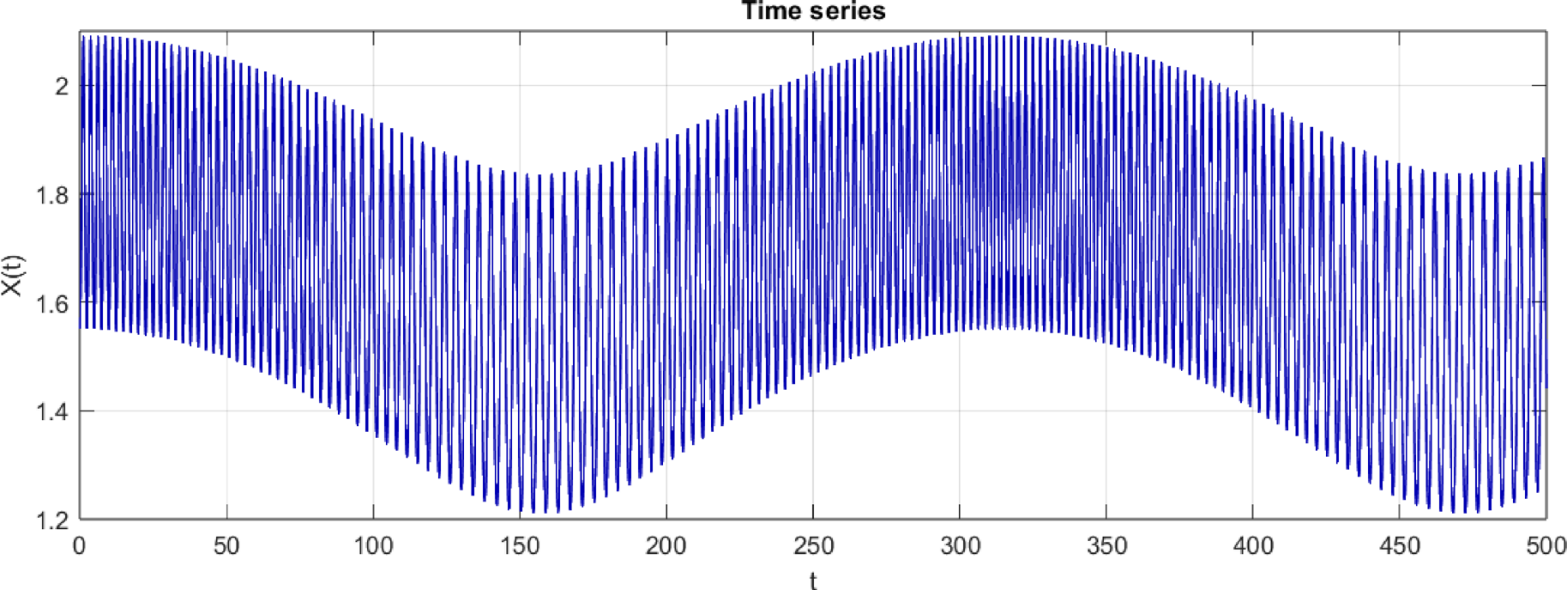
$C=3, A=0.6 b=0.02, IC=[\mathrm{1.55,0}]$ Time series only with an increased period.

**Fig. 14:**
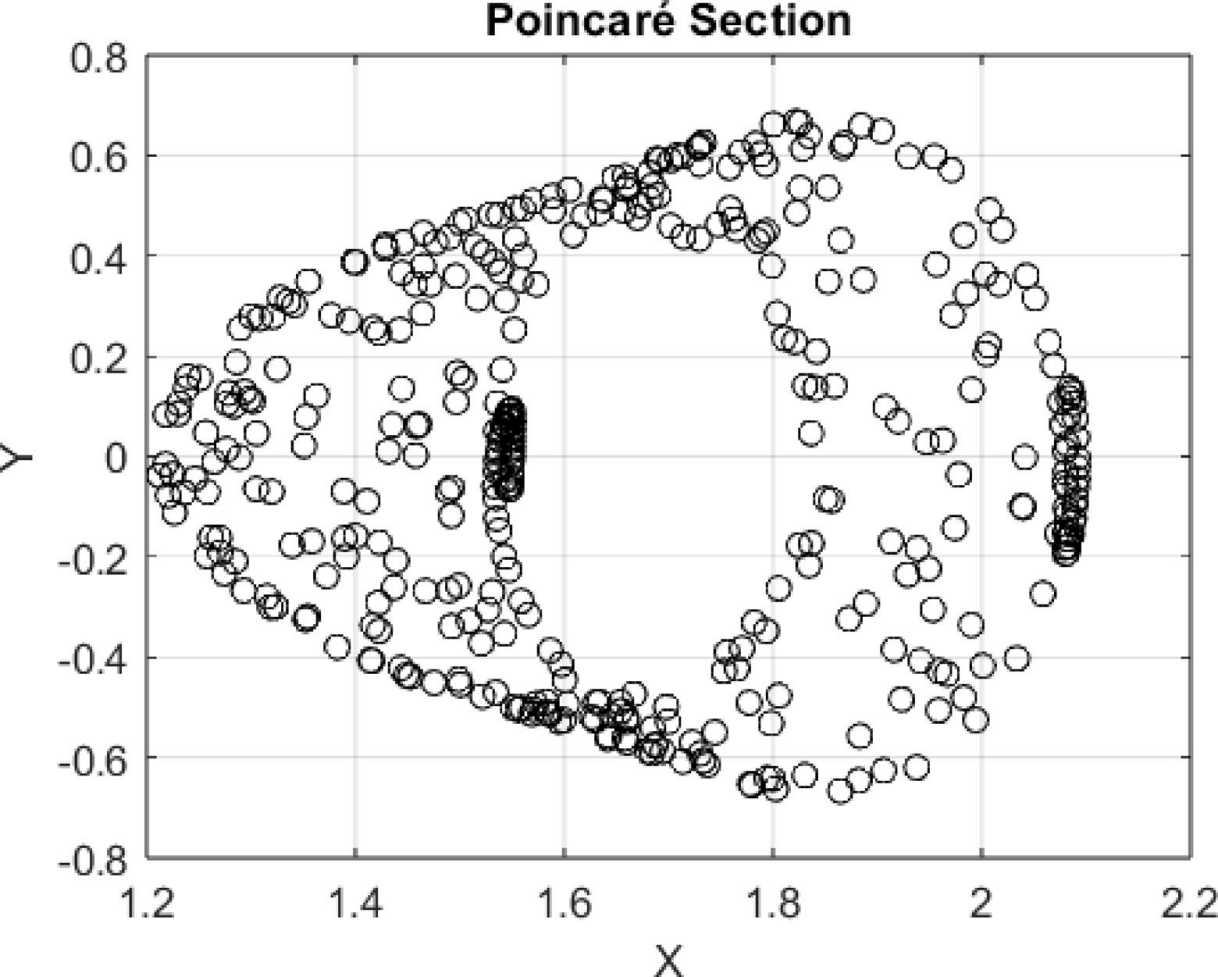
$C=3, A=0.6 b=0.02, IC=[\mathrm{1.55,0}]$, Period $T=1.25$ which filled a torus-like shape with points (therefore quasi-periodic).

In the current study, 2D phase, 3D phases, Poincaré section, and time series of Eq. (19) are analyzed. Equation (19) is exposed in Figs. 12, 13, and 14. The following properties can be obtained from these figures:

- The points are not in the form of a closed curve or discrete points.
- They are, instead, thickly packed into a torus-shaped or shell-shaped form with a structured, although non-repeating form.
- No clear periodic loop, and points do not coincide with sequential intersections.
- It exhibits quasi-periodicity because of the donut/torus-like structure and periodic bounded behavior.

Based on Fig. 13, one can observe that the number of peaks in the range of t = 0 and t = 20 is approximately 15–16, which indicates a period of $T\approx 20/16=1.25$ (Period from visible observations).Fig. 14$C=3, A=0.6 b=0.02, IC=[\mathrm{1.55,0}]$, Period $T=1.25$ which filled a torus-like shape with points (therefore quasi-periodic).Fig. 14$C=3, A=0.6 b=0.02, IC=[\mathrm{1.55,0}]$, Period $T=1.25$ which filled a torus-like shape with points (therefore quasi-periodic).Fig. 14$C=3, A=0.6 b=0.02, IC=[\mathrm{1.55,0}]$, Period $T=1.25$ which filled a torus-like shape with points (therefore quasi-periodic).

### Perturbation term of the trigonometric sin function

Let $P(t) =A sin bt$, then Eq. (18) becomes

20 $$X^{\prime } = Y\;{\mathrm{and}}\;Y^{\prime } = \frac{{\left[ {cX - X^{3} + 0.5 Y^{2} } \right]}}{{\left( {c - X} \right)}} + A\sin bt.$$

**Fig. 15:**
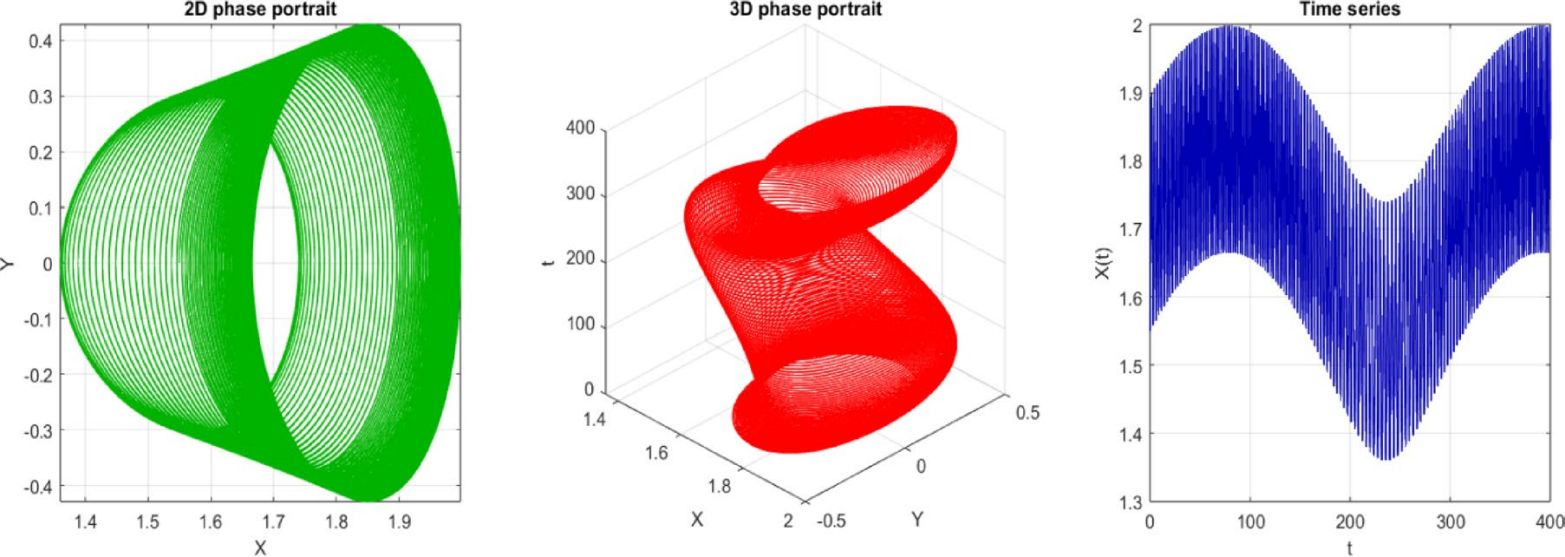
2D, 3D phase portrait and time series of the perturbed system at $c=3, A=0.6$ $b=0.02,$ with IC $=[\mathrm{1.55,0}]$.

**Fig. 16:**
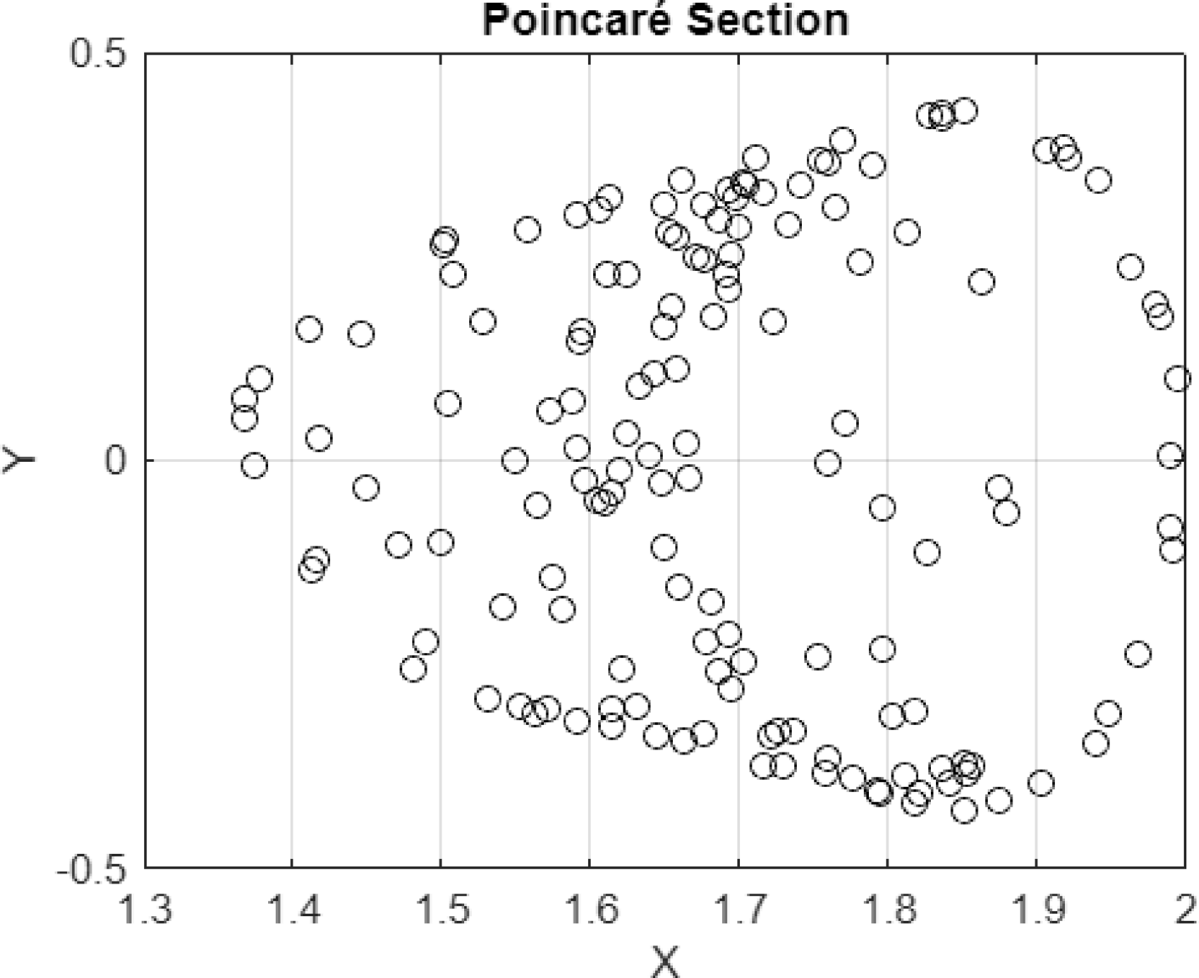
$C=3, A=0.6 b=0.02, IC=[\mathrm{1.55,0}]$, Period $T=1.25$, which filled a torus-like shape with points (therefore quasi-periodic, similar to the previous one).

In the current study, the 2D phase, the 3D phases, the Poincaré section, and the time series of Eq. (20) are pulled out. Figures 15 and 16 give the following properties of Eq. (20), which are shown:

(i) The 2D phase diagram indicates the path of the system on the X–Y plane with sinusoidal forcing. Closed loops that are smooth will show periodic or quasi-periodic motion, whereas irregular or tangled patterns will imply chaotic behavior. This visualization is useful in determining the fixed points, their stability, and the general form of the flow of the system.
(ii) The 3D phase diagram is an extension of the 2D projection to incorporate time or some other system variable. Periodic surfaces (usually called quasi-periodic) are a sign of quasi-periodicity, and non-periodic surfaces (which are twists or folds) are a sign of chaos. This model gives a more explicit image of the development that the system has undergone, as well as the impact of the forcing term.
(iii) The Poincaré section is used in sampling the system after discrete times, typically after every forced period. One or a few points imply periodic motion, smooth closed curves imply quasi-periodic behavior, and scattered points imply chaos. It gives a concise methodology for representing the long-term structure and stability of the trajectories.
(iv) Time series demonstrates how $X(t)$ or $Y\left(t\right)$ changes with time under the specified initial condition. Repeating periodic motion is characterized by regular, repeating oscillations, and quasi-periodicity is characterized by layered cycles.

### Perturbation term of Gaussian function

Let $P(t) =A {e}^{-\frac{{\left(\eta t\right)}^{2}}{2}}$, then Eq. (18) becomes

21 $$X^{\prime } = Y\;{\mathrm{and}}\;Y^{\prime } = \frac{{\left[ {cX - X^{3} + 0.5 Y^{2} } \right]}}{{\left( {c - X} \right)}} + A e^{{ - \frac{{\left( {\eta t} \right)^{2} }}{2}}} .$$

**Fig. 17:**
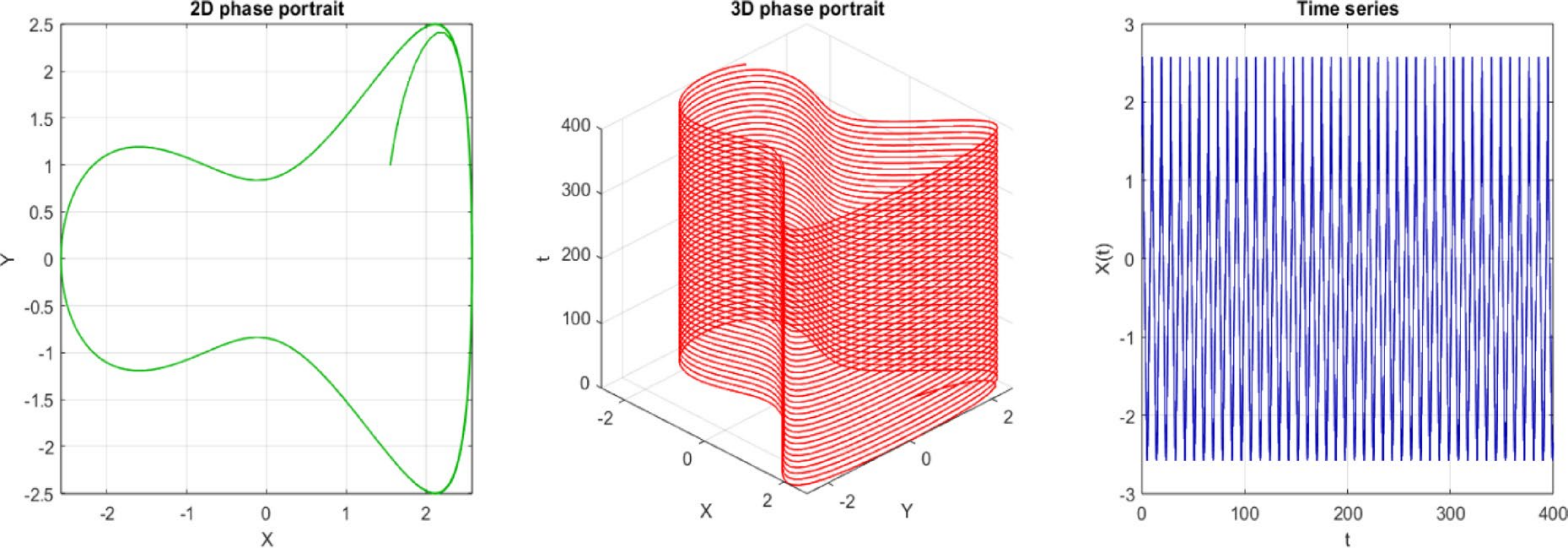
2D, 3D phase portrait and time series of the perturbed system at $c=3, A=5$ $b=5,$ with IC $=[\mathrm{1.55,1}]$.

**Fig. 18:**
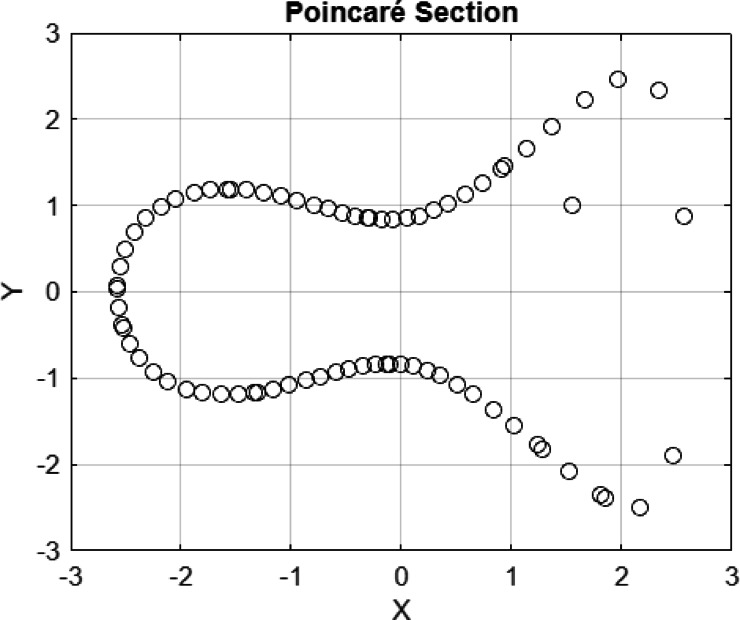
At $c=3, A=5, \eta =5, IC=[\mathrm{1.55,1}]$, Pincare map shows a smooth closed curve.

In the current work, the 2D phase diagram, the 3D phase diagram, the Poincaré section, and the time series of Eq. (21) are presented. Figures 17 and 18 give us values of the following properties of Eq. (21):

(i) The plot points are on a smooth closed curve, and this creates a clear structure that does not fill an area thickly.
(ii) There is neither scatter nor fractal structure, nor random overlap of the points.
(iii) The building is reiterated neatly in the form of a torus (looping).

Therefore, Eq. (21) is quasi-periodic.

### Perturbation term of hyperbolic function

Let $P(t) =A\mathrm{sinh}bt$, then Eq. (18) becomes

22 $$X^{\prime } = Y\;{\mathrm{and}}\;Y^{\prime } = \frac{{\left[ {cX - X^{3} + 0.5 Y^{2} } \right]}}{{\left( {c - X} \right)}} + A\sinh bt.$$

**Fig. 19:**
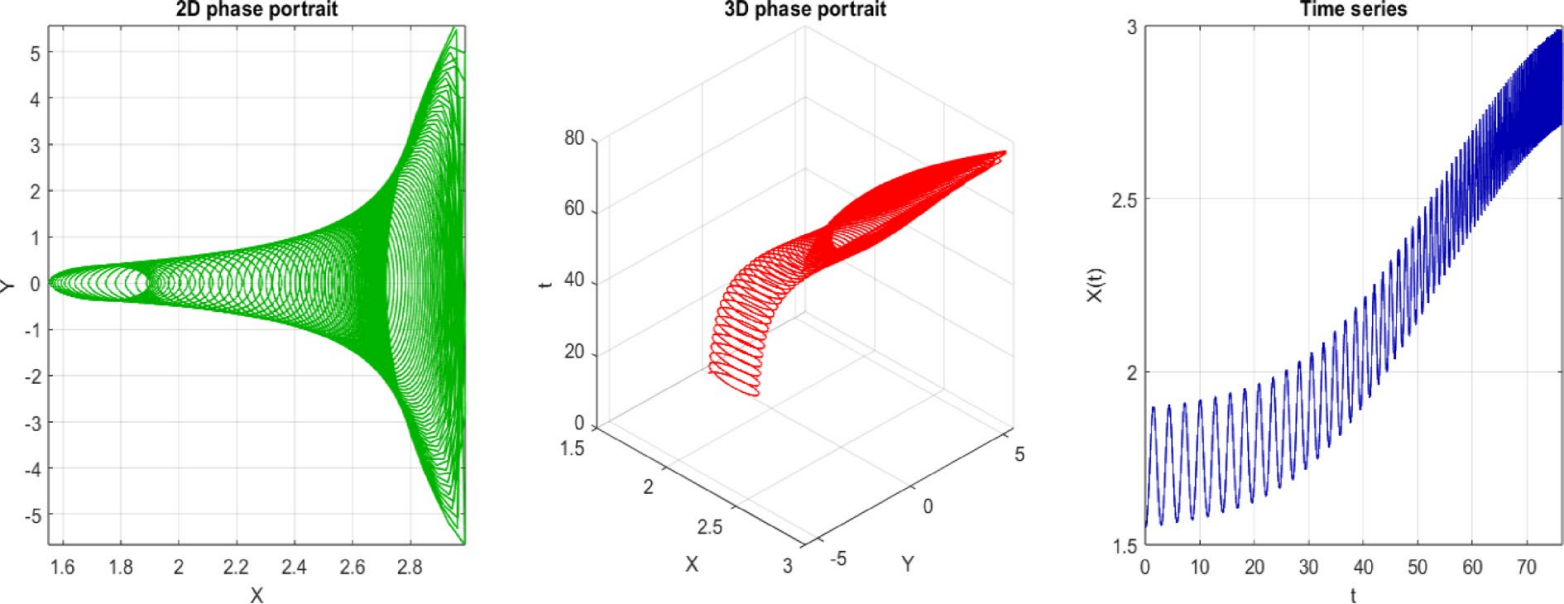
2D, 3D phase portrait and time series of the perturbed system at $c=3, A=0.1$ $b=0.1,$ with IC $=[\mathrm{1.55,0}]$.

The present study sketched a 2D phase portrait, a 3D phase portrait, a Poincaré section, and a time series of Eq. (22), which are revealed in Fig. 19. According to Fig. 19, it is apparent that the trajectory is becoming unboundedly growing in the phase plane with an increase in time. The time series also confirms the same. When the Sinh perturbation term is added to the system, the system becomes unstable. The current study visualizes the 2D phase portrait, 3D phase portrait, Poincaré section, and time series of Eq. (22) in Fig. 19. It is observed that the phase-plane trajectory increases indefinitely with time, indicating that the state of the system is divergent. This is again affirmed by the time series, which shows that the system becomes unstable after the addition of the Sinh perturbation term.

## Stability Analysis of the Obtained Solutions

This part is related to the study of the stability analysis of the attained solutions of the governing model by means of a trusted method, the linear stability method. We shall take $\Upsilon$ to be the stationary state solution of Eq. (7). In order to check its stability, we test $P\left(x, t\right)=U(\xi )$ with a perturbed wave function at their stationary states $\Upsilon$. As such, the new wave solution of the governing model of Eq. (7) is

23 $$P\left(x, t\right)=U\left(\xi \right)+{\Upsilon\Psi }\left(\xi ,t\right), 0<\Upsilon \ll 1,$$

in which $\Psi \left(\xi ,t\right)$ is a small perturbation. Substituting Eq. (23) into Eq. (7) and retaining linear terms in $\Upsilon$, we have the following linearized perturbation equation:

24 $${\Psi }_{t}-{\Psi }_{xxt}+3{\left({U}^{2}\Psi \right)}_{x}-{\left({U}{\prime}{\Psi }_{x}\right)}_{x}-{\left(U{\Psi }_{xx}\right)}_{x}-{\left(\Psi {\mathrm{U}}^{{^{\prime}}{^{\prime}}}\right)}_{x}=0$$

In order to study the temporal behaviour of the perturbation, we assume a normal-mode form:

25 $$\Psi \left(\xi ,t\right)={e}^{\Omega t}W\left(\xi \right),$$

in which $\Omega$ is the growth rate of the perturbation. Substitution of Eq. (25) into Eq. (24), the eigenvalue problem is obtained as:

26 $$\Omega \left(W-{W}^{{\prime}{\prime}}\right)+3{({U}^{2}W)}{\prime}-{\left({U}{\prime}{W}{\prime}\right)}{\prime}-{\left(U{W}^{{\prime}{\prime}}\right)}{\prime}-{\left({U}^{{\prime}{\prime}}W\right)}{\prime}=0$$

The stability of the travelling-wave solution is determined by the spectrum of $\Omega$. In case $\mathrm{Re}(\Omega )<0$, the time dependence of perturbations decreases exponentially, and the solution is stably linear. In the event of $\mathrm{Re}(\Omega )=0$, the solution is neutrally stable, but $\mathrm{Re}(\Omega )>0$ is the case of instability. Moreover, the spectral analysis can be supported by an energy-based argument. The energy of perturbation can be defined as

27 $$\mathrm{E}\left(\mathrm{t}\right)=\frac{1}{2}{\int }_{-\infty }^{\infty }({\Psi }^{2}+{\Psi }_{x}^{2})\mathrm{d}\xi ,$$

and using Eq. (24) along with conditions that allow decay to infinity, one can deduce that $\frac{dE}{dt}\le 0$ is a decreasing function that means that the perturbation energy does not increase with time. Therefore, the travelling-wave solutions obtained are stable to small perturbations in the given ranges of parameters.

## Conclusion and future work

### Conclusions

A detailed collection of precise soliton solutions, such as single singular solitons, two singular solitons, multi-solitons (bright and dark), multi-solitons (singular and multiple), and singular solutions, was obtained in the MCH equation in this work. With the help of detailed visualization (including three-dimensional, contour, density, 3D revolving, and two-dimensional time-evolution plots), the effects of different parameters on the dynamics and propagation of solitons were well illustrated. The modified (G′/G)-expansion (MG′/GE) method used in this case was highly effective to provide a wide range of 30 perfect solutions with significantly lower calculation costs than the current solutions. The derived exact soliton solutions can be of great importance in various physical and engineering systems such as optical fiber communication, plasma physics, shallow water wave dynamics, and fluid transport systems. The solutions are effective in the prediction of the wave propagation, behavior of stability, and the control of the nonlinear wave interactions in practical environments. The stability, bifurcation, and dynamical analyses also have significant implications for determining the behavior of systems on a long-term basis, identifying transitions to chaos, and developing stable wave-like systems. Comprehensively, the resulting solutions provide a solid theoretical foundation for modeling intricate nonlinear phenomena and may inform future experimental and numerical research in applied science and engineering. We further undertook the stability analysis of the obtained soliton solutions using the linear stability paradigm to illustrate the versatility of the solutions obtained. Through understanding the bifurcation theory, we examined the equilibrium and stability points of the relevant 2D dynamical system. We have used phase portraits, contours, and projections of the Hamiltonian function to demonstrate the long-term behaviour of trajectories about fixed points. We also examined bifurcations and chaotic behaviours using phase-plane analysis, which showed substantial qualitative variations in the related dynamical structures. It has enhanced our knowledge of complex nonlinearities by studying quasiperiodic and chaotic waveforms that are affected by external forces. The stability analysis also helps to corroborate the fact that the bifurcation structures and chaos dynamics described in this paper are the resultant products of the dynamically stable solution bridges.

This approach is powerful and flexible, as it offers a substantial number of solutions and is more diverse than those of previous studies. These findings underscore the theoretical and practical relevance and validity of the MG/GE method as a means of modeling nonlinear complex phenomena. More extensions to higher-dimensional systems, bringing together machine learning, numerical methods, and experimental validation in physical applications (optical fibers, plasma, and fluid dynamics), could also be a focus of future work, thus reinforcing the linkage between theory and practice.

### Future work

In this research study, the modified (G′/G)-expansion (MG′/GE) method was used to find soliton solutions to nonlinear evolution equations. This method involves a trial solution that is well selected and a support equation to convert the governing model into a system of algebraic equations. It is usually necessary to use computational software (like Mathematica, Maple, MATLAB, or Python) to solve these algebraic systems. Nevertheless, in certain situations, even sophisticated computational tools might not be able to discover exact solutions, indicating a disparity in accessibility and computability for practical use. These findings have several possible uses in a wide variety of areas, such as laser science, nonlinear optics, neural networks, artificial intelligence, and engineering. Future studies aim to refine and optimize these techniques, making them accessible and applicable to a wider range of practitioners in these fields. Moreover, the current study confirms the strength and effectiveness of the integration of chaos and multistability analysis in terms of bifurcation theory using the MG′/GE technique to obtain precise invariant solutions to time M-fractional nonlinear evolution equations. The systematic and generalizable framework of the Lie symmetry approach has more opportunities to investigate other equations of nonlinear evolution, thereby gaining a deeper understanding of their dynamical behavior. Subsequent studies can use methods to study complicated nonlinear systems, discover new soliton forms, and identify additional applications in reality, ultimately leading to innovative developments in both theoretical and practical sciences.

Future research may extend the analytical frameworks developed in this study to stochastic nonlinear models, including the higher-order stochastic modified Gerdjikov–Ivanov equation. The derivation of exact or approximate solutions for such stochastic partial differential equations (SPDEs) using advanced techniques, such as the improved modified extended tanh-function (IMETF) method, could yield deeper insight into the effects of randomness and noise on nonlinear wave propagation and soliton dynamics^45^. Another promising research direction involves the incorporation of fractional-order derivatives into nonlinear evolution equations. In particular, the retrieval and analysis of soliton solutions in birefringent optical fibers governed by conformable fractional derivatives may be investigated using robust analytical approaches. This extension would enable the accurate modelling of memory and hereditary effects inherent in complex physical media, with significant implications for optical communication systems and related technologies^46^.

Further investigations can be made into the stochastic nonlinear formulations of the theories that have already been discovered during the current research, such as the stochastic higher-order stochastic modified Gerdjikov-Ivanov equation. Advanced methods, including the improved modified extended tanh-function (IMETF) method, might be used to derive exact or approximate solutions of stochastic partial differential equations (SPDEs) and gain a better understanding of the impact of randomness and noise on nonlinear wave propagation and soliton dynamics^45^. The other research avenue that may be explored is to integrate the use of fractional-order derivatives in NLEEs. More specifically, the methods of retrieval and analysis of soliton solutions in birefringent optical fibers that are governed by conformable fractional derivatives can be explored with the help of powerful methods of analysis. This extension would allow the proper modeling of the effects of memory, as well as hereditary effects of more complex physical media, with great importance in the context of optical communication systems and technologies that utilize them^46^.

## Data Availability

Data will be made available on request from the first author, Md. Nur Alam (nuralam.pstu23@gmail.com).

## Abbreviations

DP: Degasperis–Procesi
SMCH: Simplified modified Camassa–Holm
PDEs: Partial differential equations
ODEs: Ordinary differential equations
DP: Degasperis–Procesi
CH: Camassa–Holm (CH)
MCH: Modified Camassa–Holm
MG′/GE: Modified (G′/G)-expansion method
3D: Three dimensions
2D: Two dimensions
NLEEs: Nonlinear evolution equations
KdV: Kortewegde Vries
